# N‐terminomics reveals control of Arabidopsis seed storage proteins and proteases by the Arg/N‐end rule pathway

**DOI:** 10.1111/nph.14909

**Published:** 2017-11-23

**Authors:** Hongtao Zhang, Lucy Gannon, Kirsty L. Hassall, Michael J. Deery, Daniel J. Gibbs, Michael J. Holdsworth, Renier A. L. van der Hoorn, Kathryn S. Lilley, Frederica L. Theodoulou

**Affiliations:** ^1^ Plant Sciences Department Rothamsted Research Harpenden AL5 2JQ UK; ^2^ Cambridge Centre for Proteomics Department of Biochemistry and Cambridge Systems Biology Centre University of Cambridge Cambridge, CB2 1QR UK; ^3^ Computational and Analytical Sciences Department Rothamsted Research Harpenden AL5 2JQ UK; ^4^ School of Biosciences University of Birmingham Edgbaston B15 2TT UK; ^5^ School of Biosciences University of Nottingham Loughborough LE12 5RD UK; ^6^ Plant Chemetics Laboratory Department of Plant Sciences University of Oxford Oxford OX1 3RB UK

**Keywords:** *Arabidopsis thaliana*, cruciferin, N‐end rule, N‐terminomics, protease, quantitative proteomics, TAILS, tandem mass tag (TMT)

## Abstract

- The N‐end rule pathway of targeted protein degradation is an important regulator of diverse processes in plants but detailed knowledge regarding its influence on the proteome is lacking.
- To investigate the impact of the Arg/N‐end rule pathway on the proteome of etiolated seedlings, we used terminal amine isotopic labelling of substrates with tandem mass tags (TMT‐TAILS) for relative quantification of N‐terminal peptides in *prt6*, an *Arabidopsis thaliana* N‐end rule mutant lacking the E3 ligase PROTEOLYSIS6 (PRT6).
- TMT‐TAILS identified over 4000 unique N‐terminal peptides representing *c*. 2000 protein groups. Forty‐five protein groups exhibited significantly increased N‐terminal peptide abundance in *prt6* seedlings, including cruciferins, major seed storage proteins, which were regulated by Group VII Ethylene Response Factor (ERFVII) transcription factors, known substrates of PRT6. Mobilisation of endosperm α‐cruciferin was delayed in *prt6* seedlings. N‐termini of several proteases were downregulated in *prt6*, including RD21A. RD21A transcript, protein and activity levels were downregulated in a largely *ERFVII*‐dependent manner. By contrast, cathepsin B3 protein and activity were upregulated by *ERFVII*s independent of transcript.
- We propose that the PRT6 branch of the pathway regulates protease activities in a complex manner and optimises storage reserve mobilisation in the transition from seed to seedling via control of ERFVII action.

## Abstract 2

See also the Commentary on this article by https://doi.org/10.1111/nph.15156.

## Introduction

The transitions from dormant seed to photosynthetically active plant are key steps in the life cycle of plants (Holdsworth *et al*., 2008; Wu, 2014; de Wit *et al*., 2016). Dependent on the light environment following germination, a seedling may undergo skotomorphogenesis (hypocotyl elongation in the dark) or photomorphogenesis (opening of the apical hook and development of the photosynthetic apparatus). In both cases, mobilisation of seed storage reserves fuels growth until plants become fully photoautotrophic (Penfield *et al*., 2006b; Theodoulou & Eastmond, 2012). Seed reserves comprise starch, lipids in the form of triacylglycerol (TAG) and specialised seed storage proteins (SSPs), but the relative proportions differ considerably between species (Baud *et al*., 2008). In oilseed plants, such as Arabidopsis, TAG is the most abundant storage reserve but the endosperm and embryo of Arabidopsis seeds also contain numerous protein storage vacuoles (PSVs). Arabidopsis has two major classes of SSP: the 12S globulins (cruciferins) and 2S albumins (napins) which are synthesised as precursors during seed maturation and accumulate in PSVs after processing (Herman & Larkins, 1999; Baud *et al*., 2008). Following imbibition, catabolism of lipid and protein reserves is initiated in endosperm cells adjacent to the radical tip (Mansfield & Briarty, 1996). Tissue‐specific analysis of abscisic acid (ABA) signalling has shown that mobilisation of embryo and endosperm lipid reserves is under distinct hormonal control (Penfield *et al*., 2004, 2006a).

**Figure 1:**
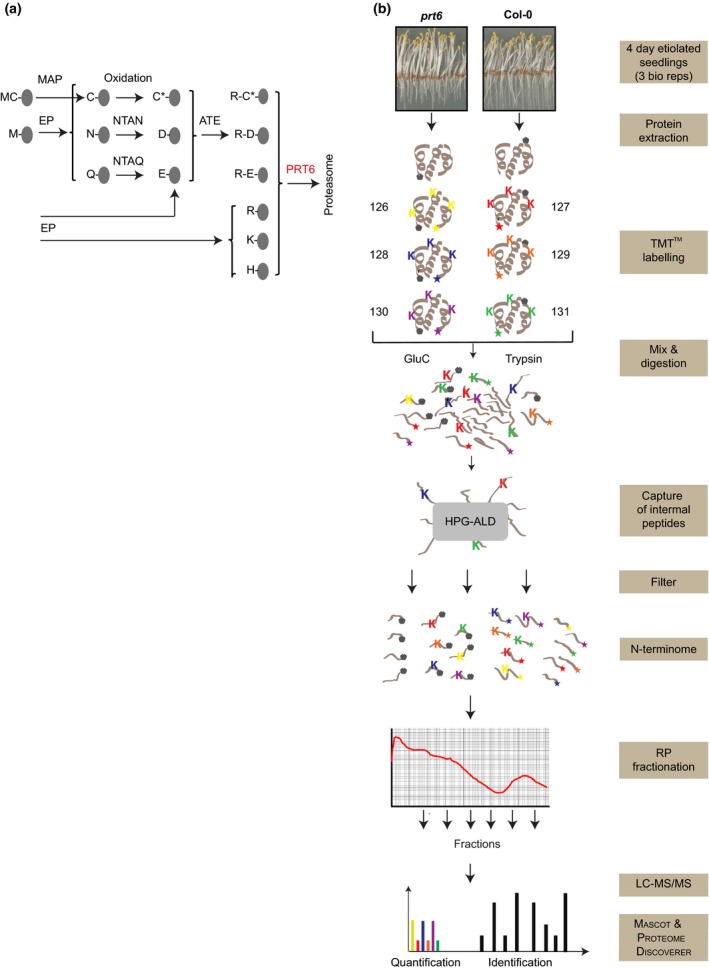
Identification and quantitation of N‐terminal peptides with TMT™‐TAILS. (a) The PRT6 branch of the Arg/N‐end rule. Substrates are generated by the action of endopeptidases (EP) or by methionine aminopeptidase (MAP)‐dependent excision of Met1 from proteins initiating Met‐Cys. PRT6, PROTEOLYSIS6 E3 ligase; ATE, arginyl tRNA transferase; NTAN1, asparagine‐specific N‐terminal amidase; NTAQ1, glutamine‐specific N‐terminal amidase. Amino acids are indicated with single letter codes; C*, oxidised cysteine. (b) Schematic representation of the TAILS workflow. Primary amines of proteins with free N‐termini (star) and lysine (K) side‐chain amines of proteins were labelled with 6‐plex TMT reagents (three biological replicates per genotype). After combining labelled samples from WT and *prt6‐5* plants, the sample was divided into two, proteins were digested with either GluC or trypsin, and internal peptides were removed via hyperbranched polyglycerol aldehyde (HPG‐ALD) polymer binding of the free N‐terminal amine group. The unbound peptides (highly enriched for N‐terminal peptides) were fractionated by reversed‐phase (RP) chromatography, then analysed by high‐accuracy LC‐MS/MS. Mascot and ProteomeDiscoverer™ were used for protein identification and quantification. Grey pentagons represent naturally blocked (acetylated) N‐termini.

Numerous genetic studies have provided valuable insight into the control of germination and seedling establishment (Holdsworth *et al*., 2008). Previously, we identified *PROTEOLYSIS6* (*PRT6*) as a positive regulator of germination in Arabidopsis (Holman *et al*., 2009). *prt6* null alleles exhibit a range of phenotypes related to germination and seedling establishment: germination of *prt6* is hypersensitive to inhibition by ABA and insensitive to nitric oxide (NO), *prt6* seedling establishment is hypersensitive to sucrose, and hypocotyls and endosperm of *prt6* seedlings retain oil bodies for several days following germination (Holman *et al*., 2009; Gibbs *et al*., 2014a). *PRT6* encodes a ubiquitin E3 ligase belonging to the N‐end rule pathway of targeted protein degradation, which is a specialised subset of the ubiquitin proteasome system (Bachmair *et al*., 1986; Garzón *et al*., 2007; Varshavsky, 2011; Gibbs *et al*., 2014b, 2016). The N‐end rule relates the half‐life of a protein to its amino terminal (Nt) residue and has three branches, the Arg/N‐end rule and the Ac/N‐end rule, which target free and acetylated N‐termini, respectively, and the recently defined Pro/N‐end rule pathway (Supporting Information Fig. S1; Hwang *et al*., 2010; Varshavsky, 2011; Chen *et al*., 2017). In eukaryotes, proteins are synthesised with Met at the N‐terminus but can become Arg/N‐end rule substrates following cleavage by nonprocessive endopeptidases, if the new Nt is large or bulky (a so‐called destabilising residue). Arabidopsis has two characterised E3 ligases that recognise different types of destabilising residues. PROTEOLYSIS1 (PRT1) recognises aromatic Nt amino acids, whereas PRT6 is specific for basic Nt residues (Potuschak *et al*., 1998; Stary *et al*., 2003; Garzón *et al*., 2007; Graciet *et al*., 2010; Mot *et al*., 2018). As well as primary destabilising residues revealed by endopeptidase cleavage, PRT6 substrates can be generated via enzymatic modification of secondary and tertiary destabilising residues (Figs 1, S1). Five Arabidopsis transcription factors belonging to Group VII of the Ethylene Response Factor (ERFVII) family, namely HYPOXIA RESPONSIVE1 (HRE1), HRE2, RELATED TO APETALA2.2 (RAP2.2), RAP2.3 and RAP2.12, are N‐end rule substrates (Gibbs *et al*., 2011, 2015; Licausi *et al*., 2011). These proteins are substrates by virtue of a Cys residue at position 2: following N‐terminal Met excision (NME), Cys2 is oxidised by specific oxidases, which enables Nt arginylation, catalysed by arginyltransferase enzymes, ATE1 and ATE2. The sequential reactions of NME, Cys oxidation and arginylation produce an Nt degradation signal (N‐degron) for PRT6. The stability of these Met‐Cys initiating transcription factors is controlled by oxygen availability and action on exposed Cys‐2, thereby providing a mechanism by which oxygen status is sensed and transduced by the Arg/N‐end rule pathway in plants (Gibbs *et al*., 2011, 2015; Licausi *et al*., 2011; Weits *et al*., 2014; Mendiondo *et al*., 2016; White *et al*., 2017). Hypoxia responsive genes, such as *ALCOHOL DEHYDROGENASE* (*ADH*), *PYRUVATE DECARBOXYLASE* (*PDC*) and *HAEMOGLOBIN1*, are ectopically expressed in *prt6* alleles (Choy *et al*., 2008; Gibbs *et al*., 2011; Riber *et al*., 2015). The Arg/N‐end rule also acts as a sensor of NO, which is required in addition to O_2_ for the degradation of ERFVII proteins in plants and G‐protein regulators in mammals (Hu *et al*., 2005; Gibbs *et al*., 2014a, 2015).

Genetic approaches in Arabidopsis have revealed further roles for the PRT6 branch of the Arg/N‐end rule pathway in leaf development and senescence (Yoshida *et al*., 2002; Graciet *et al*., 2009), quiescence under submergence (Riber *et al*., 2015), plant–pathogen interactions (Gravot *et al*., 2016; de Marchi *et al*., 2016) and photomorphogenesis (Choy *et al*., 2008; Abbas *et al*., 2015). The Arg/N‐end rule also plays roles in gametophyte development, starch accumulation and senescence in the moss *Physcomitrella patens* (Schuessele *et al*., 2016). With the exception of germination, gas sensing and photomorphogenesis, which are ERFVII‐dependent (Gibbs *et al*., 2014a,b; Abbas *et al*., 2015), the mechanisms underlying N‐end rule loss of function phenotypes have not been identified. In this study, we set out to determine the impact of the PRT6 E3 ligase on the proteome. We hypothesised that substrates would be stabilised in the *prt6* mutant and therefore increased in abundance relative to the wild type, as would proteins acting downstream of PRT6 substrates such as transcription factors. Quantitative proteomics techniques, in particular N‐terminome analysis (Huesgen & Overall, 2012; Tsiatsiani *et al*., 2012), offer an opportunity to analyse the N‐end rule in this way: enrichment of N‐terminal peptides not only simplifies the proteome but also provides information about protein cleavage events that can be used to identify and validate potential N‐end rule substrates (Kleifeld *et al*., 2010, 2011). The N‐terminome is also a useful resource for protein annotation (Hartmann & Armengaud, 2014; Lange *et al*., 2014; Willems *et al*., 2017). Previously, we achieved efficient enrichment of Nt peptides from roots of Arg/N‐end rule mutants, using terminal amine isotopic labelling of substrates (TAILS) coupled with dimethyl labelling (Zhang *et al*., 2015). Here, we incorporate tandem mass tag (TMT™) labelling into the TAILS workflow to quantify the impact of the Arg/N‐end rule on etiolated seedlings. We identified and quantified *c*. 4000 Nt peptides. Of these, Nt peptides corresponding to 146 protein groups exhibited significantly altered abundance in *prt6* seedlings. Surprisingly, we detected increased levels of SSP N‐termini in *prt6*, notably representing all four major cruciferins. We provide evidence that this reflects delayed mobilisation in Arg/N‐end rule mutants, due to increased stability of the ERFVII transcription factors. Our N‐terminomics data set also revealed that several proteases were differentially regulated in *prt6*, and subsequent validation showed that protease accumulation and activity are subject to complex regulation by the ERFVIIs. Collectively, our studies reveal that the Arg/N‐end rule serves to co‐ordinate the mobilisation of seed storage reserves and to regulate the abundance and activities of several proteases following germination.

## Materials and Methods

### N‐end rule mutant alleles and transgenic lines

*prt6‐1*,* prt6‐5* and *ate1/2* are well‐characterised *Arabidopsis thaliana* L. Heynh. null T‐DNA alleles, described by Holman *et al*. (2009) and Graciet *et al*. (2009). Higher order mutants are described by Gibbs *et al*. (2011, 2014a) and Abbas *et al*. (2015). X‐GUS lines are described by Garzón *et al*. (2007).

### Plant growth and seedling treatments

Seeds were raised from plants grown under long day conditions (16 h : 18 h; 23°C : 18°C); all genotypes to be compared were raised in the same cabinet. Seeds were harvested, sieved (< 425 µm; Endecotts, London, UK ) and stored at room temperature. After ripened seeds were surface‐sterilised and plated on nylon mesh (Sefar NITEX, 03‐110/47; Heiden, Switzerland) on 0.5× Murashige and Skoog (MS) medium containing 0.5% (w/v) sucrose. After 2–3 d dark chilling at 4°C, plates were exposed to light for 6 h to induce germination, then wrapped in foil and incubated in a vertical position at 22°C for 4 d. Etiolated seedlings were harvested under green light; note that mutant and wild type (WT) seedlings grown under these conditions were at the same developmental stage.

### TMT labelling and enrichment of N‐termini by TAILS

TMT‐TAILS was performed according to Klein *et al*. (2015) and Prudova *et al*. (2016), with modifications and MS as described in Methods S1.

### TAILS MS data analysis

Raw data were searched against the TAIR10 database using Mascot v.2.4 (Matrix Science, London, UK) and Proteome Discoverer™ v.1.4.1.14 as described by Zhang *et al*. (2015), employing Top 10 peaks filter node and percolator nodes and reporter ions quantifier with semi‐ArgC or semi‐GluC enzyme specificity with a maximum of one missed cleavage. Carbamidomethylation (+57.021 Da) of cysteine and TMT isobaric labelling (+229.162 Da) of lysine were set as static modifications while TMT (+229.162 Da) labelling of the peptide N‐termini, the acetylation of the peptide (+42.011) N‐termini and methionine oxidation (+15.996) were considered dynamic. Mass tolerances were set to 10 ppm for MS and 0.06 Da for MS/MS. For quantification, integration window tolerance was set to 0.0075 Da. Each reporting ion was divided by the sum of total ions. Ratios were normalised by the medians of pre‐TAILS samples (Methods S1; Lange *et al*., 2014) searched with ArgC or GluC specificity. Statistical significance of quantification was assessed with an unpaired two‐sample Student's *t*‐test on 4 df. Data were log transformed and statistically significant results (*P *>* *0.05) were further restricted to those with more than two‐fold change. No correction for multiplicity was applied. The statistical software package R 3.2.2 was used for all analyses. MS proteomics data have been deposited to the ProteomeXchange Consortium via the PRIDE partner repository (Vizcaíno *et al*., 2016) with the dataset identifier PXD006450.

### SDS‐PAGE and immunoblotting

Proteins were extracted in modified RIPA buffer containing 50 mM HEPES‐KOH pH 7.8, 100 mM KCl, 5 mM EDTA, 5 mM EGTA, 50 mM NaF, 10% (v/v) glycerol, 1% (v/v) IGEPAL, 0.5% (w/v) deoxycholate, 0.1% (w/v) sodium dodecyl sulphate (SDS), 1 mM Na_4_VO_3_, 1 mM phenylmethylsulfonylfluoride, 1× proteinase inhibitor cocktail (Roche), 1× phosphostop (Roche) and 50 μM MG‐132. Proteins were separated in precast 4–12% Bis‐Tris gels, using 1× SDS MES buffer and stained with Coomassie Brilliant Blue or transferred to polyvinylidene fluoride using iblot dry blotting system (ThermoFisher, Waltham, MA, USA). Detailed information is given in Methods S2. Primary antibodies were: *Brassica napus* Cruciferin (Wan *et al*., 2007), 1 : 10 000–20 000; OLE1 (anti‐rS3; D'Andréa *et al*., 2007), 1 : 5000–10 000; Anti N‐terminal AtCathB3 (kind gift of Dr Patrick Gallois, University of Manchester), 1 : 1000; Arabidopsis RD21 (residues 137–150; LPESIDWRKKGAVAC; Kaschani *et al*., 2009; kind gift of Prof. Carol Mackintosh, Dundee), 1 : 1000; and PDC and ADH (Agrisera, Vännäs, Sweden), 1 : 10 000 and 1 : 3000. For analysis of dry seeds and dissected endosperm, identical numbers of similar size seeds or endosperm were collected using a dissecting microscope, processed in parallel and identical amount of extracts were loaded, as indicated in the figure legends.

### Real time quantitative reverse‐transcription PCR (RT‐qPCR)

Four‐day‐old etiolated seedlings without endosperm or seed coat were harvested under green light and RNA were extracted using an RNeasy Plant Mini Kit (Qiagen) and treated with RQ1 RNase‐free DNase (Promega). A Transcriptor First Strand cDNA Synthesis Kit (Roche) and anchored ‐oligo(dT)_18_ were used for cDNA synthesis for a two‐step RT‐PCR. Faststart Essential DNA Green Master (Roche) was used for real‐time PCR using a Lightcycler^®^96. Relative quantification was done using both ACT2 (At3g18780.2) and TUB4 (At5g44340.1) as references. Student's *t*‐test was used to calculate *P* values; error bars are shown as standard errors. Primers used are given in Table S1.

### Activity‐based protein profiling

Activity‐based protein profiling (ABPP) was carried out as described by Lu *et al*. (2015). Band intensities were quantified using ImageJ. Student's *t*‐test was used to calculate *P‐*values.

## Results

### The seedling N‐terminome: identification of Nt peptides by TMT‐TAILS

*prt6* RNA is expressed at a low level throughout the plant (Schmid *et al*., 2005; Winter *et al*., 2007; Zhang *et al*., 2015) and *prt6* alleles exhibit phenotypes throughout development, including the transition from dark‐grown seedlings to light (Abbas *et al*., 2015). Etiolated seedlings were selected for analysis because PRT6 is active at this developmental stage, as demonstrated by stabilisation of the artificial Arg/N‐end rule substrate, R‐GUS, in the *prt6* mutant background (Fig. S2). Labelling of proteins with TMTsixplex™ reagents was used in combination with TAILS to identify and quantify Nt peptides in seedlings of Col‐0 and the null mutant, *prt6‐5* (Graciet *et al*., 2009). The experimental workflow is presented in Fig. 1.

**Figure 2:**
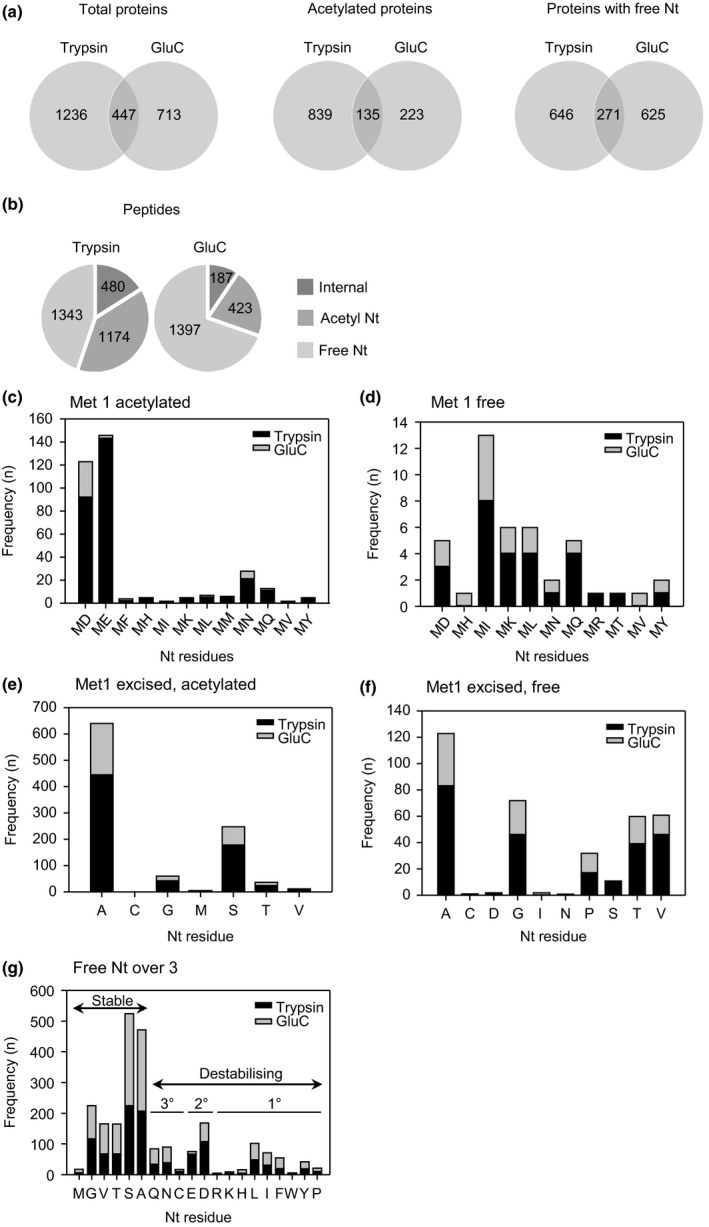
Analysis of protein groups and N‐terminal peptides identified by TMT™‐TAILS. Peptides were enriched by TMT‐TAILS, using two different proteases, trypsin and GluC. (a) Venn diagrams showing overlap in protein groups identified with the N‐terminal peptide datasets from two different proteases. (b) Numbers of unique peptides with location information identified in different categories (free N‐terminal (Nt), acetylated Nt and non‐Nt (internal) peptides) following enrichment by TAILS. When an N‐terminal peptide matched to more than one protein group, positional information was derived for the master protein defined by ProteomeDiscoverer. (c) Analysis of first and second residues of Nt peptides with Met 1 acetylated. (d) Analysis of first and second residues of Nt peptides with free Met 1. (e) Nt peptides resulting from N‐terminal methionine excision followed by Nt acetylation. (f) Free Nt peptides resulting from N‐terminal methionine excision. (g) Occurrence of different Nt‐amino acid residues in free Nt peptides which initiate at amino acid residues ≥ 3, relative to the protein encoded by the published open reading frame (ORF). Met, Gly, Val, Thr, Ser and Ala are stabilising residues. Primary, secondary and tertiary destabilising residues are indicated on the graph.

The full N‐terminome dataset for etiolated seedlings is presented in Table S2. A total of 2396 protein groups were identified, with < 20% overlap between the two proteases used in the TAILS workflow (Fig. 2a). The combined GluC and Trypsin TAILS data sets comprised 5004 unique peptides for which location information was available. Of these, 32% were acetylated, 55% had free N‐termini and the remainder represented internal peptides not removed by the hyperbranched polyglycerol aldehyde polymer (Fig. 2b). In total, 4337 unique Nt peptides representing 3648 unique N‐termini were identified. More unique peptides were identified in the tryptic digest (2997, compared to 2007 for GluC), whereas GluC yielded a higher percentage of free Nt peptides, with a lower proportion of acetylated peptides due to lack of the basic residues (Biniossek & Schilling, 2012). The majority of acetylated Nt peptides were acetylated at Met1 or at residue 2, and therefore are probably the result of co‐translational Nt acetylation, either at the original N‐terminus or following NME by Met amino peptidases (Fig. 2c,e). We also detected free N‐termini generated by NME (Fig. 2f); both these and the acetylated peptides conformed to the established specificity of Met aminopeptidases (Bonissone *et al*., 2013). Of 2740 free Nt peptides, 43 corresponded to unmodified protein N‐termini initiating with Met (Fig. 2d). The remainder of the non‐acetylated peptides putatively generated by a post‐translational cleavage event were classified as ‘neo’ Nt peptides. In total, 2332 peptides initiated at residue 3 or beyond, relative to the predicted translation start (‘other’) (Fig. 2g). As we observed previously (Zhang *et al*., 2015), peptides with destabilising residues were underrepresented in the N‐terminome.

### TMT‐TAILS identifies protein N‐termini with altered abundance in *prt6*

**Figure 3:**
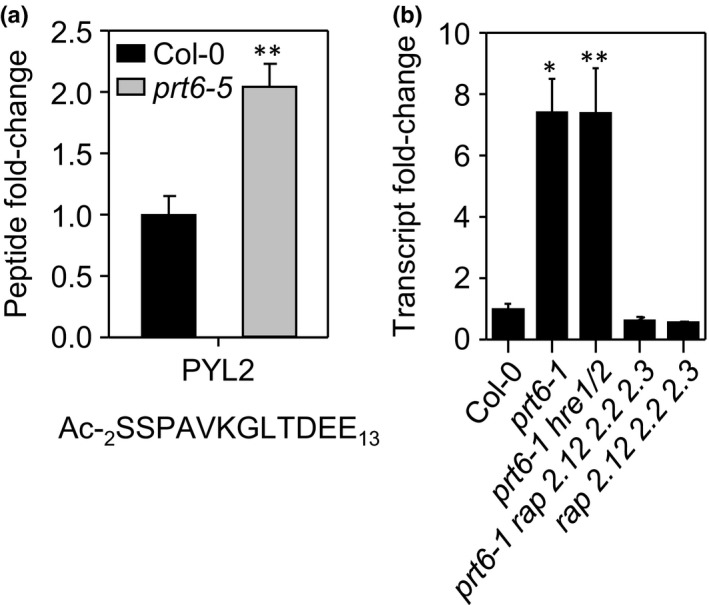
Abscisic acid (ABA) receptor component, PYL2, is regulated by the Arg/N‐end rule in an ERF‐dependent manner in etiolated *Arabidopsis thaliana* seedlings. (a) Relative abundance of Nt peptide corresponding to amino acids 2–13 of PYL2 in Col‐0 and *prt6‐5*. (b) *PYL2* transcript abundance in single and combination N‐end rule and *erfVII* mutants, relative to Col‐0. Values are means ± SE (*n *=* *3); *, *P *<* *0.05; **, *P *<* *0.01.

Peptide abundance was quantified and normalised with three biological replicates of Col‐0 and *prt6*. The majority of peptides were of similar abundance in Col‐0 and *prt6* seedlings (Fig. S3; Table S3). However, Nt peptides corresponding to 45 protein groups exhibited significantly increased abundance in *prt6* (defined as two‐fold at *P *<* *0.05; Table 1). Sixteen groups are represented only by ‘original’ N‐termini (i.e. Met 1 or residue 2, relative to the TAIR10 gene model), 27 were identified only from N‐termini generated by endopeptidase cleavage and two were represented by both the original N‐terminus and a new N‐terminus generated by cleavage. Whilst the abundance of Nt peptides may not accurately reflect the abundance of the full‐length protein, three classes of protein of particular interest with regard to the known physiological functions of *PRT6* were identified and selected for further study. First, an Nt peptide derived from the ABA receptor component, PYR1‐like 2 (PYL2), was upregulated in *prt6* (Fig. 3a). PYL2 is unlikely to be a PRT6 substrate, because the peptide did not bear an Nt destabilising residue but appeared to have been generated by NME followed by acetylation at position 2. Analysis of transcript abundance by RT‐qPCR indicated that *PYL2* expression was increased in *prt6* relative to Col‐0 and that this increase was dependent upon the ERFVII transcription factors, RAP2.12, RAP2.2 and RAP2.3, but not HRE1 and HRE2 (Fig. 3b). Eight proteins encoded by genes known to be transcriptionally upregulated by hypoxia showed increased abundance in *prt6*; these included proteins encoded by ‘core’ hypoxia responsive genes, *ADH*,* HAEMOGLOBIN1* and *ACC OXIDASE 1* (Mustroph *et al*., 2009), and also two proteins belonging to the adenine nucleotide α‐hydrolase superfamily, which are homologous to the hypoxia‐responsive universal stress protein, HRU1 (Gonzali *et al*., 2015). Remarkably, seed storage proteins represented the largest category of proteins with greater abundance in *prt6* compared to Col‐0 (Table 1). All four major 12S globulins (cruciferins) were represented by several neo‐Nt peptides in the *prt6*‐up data set.

**Table 1:** N‐terminal peptides with increased abundance in seedlings of the *Arabidopsis thaliana prt6* mutant

AGI code	Description	Synonyms	Peptide	Start	Finish	Log_2_ fold change
Seed storage proteins						
AT1G03880.1	Cruciferin 2	CRU2, CRB, At12S3	gEGQGQGQSQGFR	117	129	5.08
			qGQGQSQGFR	120	129	5.02
			gQGQGQSQGFR	119	129	4.75
			qGQSQGFR	122	129	4.65
			gQGQSQGFR	121	129	3.93
			eGQGQGQSQGFR	118	129	3.80
			gLEETLcTMR	270	279	3.63
			gQGQGQSQGFRD	119	130	3.26
			nLDDPSDAD	283	291	3.02
			gLEETLcTmR	270	279	2.65
			aLEPSQIIkSE	37	47	2.61
AT1G03890.1	RmlC‐like cupins superfamily protein	At12S2	aPFPNAcHFSQ	30	40	4.34
			aPFPNAcHFS	30	39	3.26
			eAPFPNAc	29	36	2.89
			gIEETYcTAkIHENIDDPER	271	290	2.75
			pETFAEVEGSSGR	113	125	2.17
			aPFPNAcH	30	37	2.00
			sLAPAQATkFE	43	53	1.41
AT1G52690.1	Late embryogenesis abundant protein (LEA) family protein	LEA7	aSHQEQSYkAGETR	Ac‐2	15	2.85
AT2G28490.1	RmlC‐like cupins superfamily protein		gEGEGGGEWGGGGEGGGGGR	63	82	3.68
AT3G15670.1	Late embryogenesis abundant protein (LEA) family protein		tAQSAkE	75	81	1.48
			aSNQQSYkAGETR	Ac‐2	14	1.40
AT3G22640.1	Cupin family protein	PAP85	qEEEEDmSENVHkVVSR	364	380	4.23
			qEEEEDMSENVHkVVSR	364	380	4.14
			eEEEDMSENVHkVVSR	365	380	3.52
			ePPQQGEQEGPR	33	44	2.96
			eEEEDmSENVHkVVSR	365	380	2.64
AT4G27170.1	Seed storage albumin 4	SESA, At12S4	gQQHQPEQVR	125	134	2.28
AT4G28520.1	Cruciferin 3	CRU3, CRC, At12S1	gQPWEGQGQQGQQGFR	175	190	4.84
			vGVSVARYVIE	71	81	4.81
			gQQGQQGFR	182	190	4.71
			eILYcTGGQGR	403	413	4.55
			eGQGQQGQQGFR	179	190	4.28
			dNLDVLQATE	40	49	4.15
			qQGQQGFR	183	190	4.02
			gQGQQGQQGFR	180	190	3.16
			qQGQPWEGQGQQGQQGFR	173	190	3.00
			tIcSMRSHE	338	346	2.98
			nLDNLDVLQATE	38	49	2.67
			qGQQGQQGFR	181	190	2.54
			gQGQQGQQGFR	180	190	2.37
			gLEETIcSMR	334	343	2.19
			sVNSYTLPILE	366	376	2.18
			gLEETIcSmR	334	343	1.98
			rQSLGVPPQLQNE	24	36	1.91
			gVPPQLQNE	28	36	1.59
			gQGQQGQQGFRD	180	191	1.09
			aMVLPkYNMNANE	391	403	1.09
AT5G44120.3	Cruciferin 1	CRU1, CRA1, At12S4	gLEETIcSARcTDNLDDPSR	283	302	5.22
			tDNLDDPSR	294	302	5.16
			sGVSFARYIIE	70	80	4.66
			aLEPSHVLkSE	43	53	3.65
			eTFQDSSEFQPR	114	125	3.63
			qGQQGQQFPNE	25	35	3.58
			gQQFPNEcQLDQLNALEPSHVLkSEAGR	29	56	3.55
			cTDNLDDPSR	293	302	3.38
			gLEETIcSAR	283	292	2.90
			tTLTHSSGPA	453	462	2.77
			gNNPQGQVWLQGRE	195	208	2.77
			qQGQQFPNEcQLDQLNALEPSHVLkSEAGR	27	56	2.77
			qQFPNEcQLDQLNALEPSHVLkSEAGR	30	56	2.56
			gQQGQQFPNE	26	35	2.46
			qQGQQFPNE	27	35	2.12
			fEGQGQSQR	126	134	2.00
			aETFQDSSEFQPR	113	125	1.70
			dGEAQIQIVNDNGNR	358	372	1.69
			qGQQFPNE	28	35	1.54
			tTLTHSSGPAS	453	463	1.40
			sGDTIATTPGVAQW	147	160	1.22
Hypoxia‐responsive						
AT1G43800.1	Plant stearoyl‐acyl‐carrier‐protein desaturase family protein	FTM, SAD6	gTIAADEkR	248	256	2.71
AT1G77120.1	Alcohol dehydrogenase 1	ADH1	aVGLGAAEGAR	205	215	2.09
			sTTGQIIRckAAVAWE	Ac‐2	17	1.64
AT2G16060.1	Haemoglobin 1	HB1	mESEGkIVF	Ac‐1	9	3.26
AT2G19590.1	ACC oxidase 1	AtACO1	lQDDQVPGLE	192	201	2.41
AT2G47710.1	Adenine nucleotide alpha hydrolases‐like superfamily protein		aTGDGkSVmVVGVDDSEQSTY	Ac‐2	22	1.96
AT3G11930.3	Adenine nucleotide alpha hydrolases‐like superfamily protein		aEEQAATAmETSAVEkQPE	Ac‐2	20	1.25
AT3G21720.1	Isocitrate lyase	ICL	iMEEEGR	11	17	2.30
			aVSEHINR	223	230	1.09
AT5G19550.1	Aspartate aminotransferase 2	ASP2	aDSPAITESR	89	98	1.34
Other						
AT1G06680.1	Photosystem II subunit P‐1	PSBP‐1	aQQSHEDDNSAVSR	42	55	4.11
			kAQQSHEDDNSAVSR	41	55	3.02
AT1G07600.1	Metallothionein 1A	MT1A, ATMT‐2, ATMT‐Q, LSR4	ADSNcGcGSSckcGD	2	16	1.04
AT1G14950.1	Polyketide cyclase/dehydrase and lipid transport superfamily protein		aTSGTYVTEVPLkGSAkNHY	Ac‐2	21	1.66
			aTSGTYVTEVPLkGSAkN	Ac‐2	19	1.29
AT1G17810.1	Beta‐tonoplast intrinsic protein	BETA‐TIP	eATHPDSIR	16	24	4.40
			dEATHPDSIR	15	24	2.18
			aDEATHPDSIR	14	24	1.95
AT1G23870.1	Trehalose‐phosphatase/synthase 9	TPS9	tVPGIISELDGGYSDGSSDVNSSNSSR	32	58	1.98
AT1G48130.1	1‐Cysteine peroxiredoxin 1	PER1	pGITLGDTVPNLE	2	14	1.08
AT1G54870.1	NAD(P)‐binding Rossmann‐fold superfamily protein	ChlADR	iEEIDEPR	185	192	2.22
AT1G64970.1	Gamma‐tocopherol methyltransferase	G‐TMT, VTE4, TMT1	aATSTEALR	53	61	1.11
AT1G65090.2	Unknown protein		sQTmEEYQSNESEDkR	Ac‐2	17	1.81
AT1G69410.1	Eukaryotic elongation factor 5A‐3	ELF5A‐3	sDDEHHFESSDAGASkTYPQ	Ac‐2	21	1.08
AT2G17200.1	Ubiquitin family protein	DSK2	gGEGDSSQPQSGEGEAVAVN	2	21	1.64
AT2G23240.1	Plant EC metallothionein‐like protein	AtMT4b	aDTGkGSASAScNDR	2	16	2.54
AT2G26040.1	PYR1‐like 2	PYL2	sSSPAVkGLTDE	Ac‐2	13	1.05
AT2G30950.1	FtsH extracellular protease family	VAR2, FTSH2	dEQGVSSSR	83	91	1.05
AT2G38400.2	Alanine:glyoxylate aminotransferase 3	AGT3	dSDEFQAR	35	42	1.92
AT3G13120.1	Ribosomal protein S10p/S20e family protein		dTLDPTPE	60	67	3.23
AT3G21380.1	Mannose‐binding lectin superfamily protein		aAATMSWDDGkH	Ac‐2	13	3.21
			aAATmSWDDGkH	Ac‐2	13	2.68
AT3G51100.1	Unknown protein		nEGSSEEVTR	2	11	1.00
AT3G57560.1	*N*‐Acetyl‐l‐glutamate kinase	NAGK	tVSTPPSIATGNAPSPDYR	51	69	1.28
AT3G58450.1	Adenine nucleotide alpha hydrolases‐like superfamily protein		mETYVDAIGEDTAATTTTAETAANkN	Ac‐1	26	1.58
AT3G61870.1	Unknown		aGGEFGILEGR	75	85	1.03
AT4G12420.1	Cupredoxin superfamily protein	SKU5	aDPYSFYNFE	21	30	1.25
AT4G26870.1	Class II aminoacyl‐tRNA and biotin synthetases superfamily protein		sSNYGDVTTNE	53	63	1.86
AT5G10160.1	Thioesterase superfamily protein		eIPIELR	61	67	2.64
AT5G47110.1	Chlorophyll A‐B binding family protein	LIL3:2	aSSDNGTTSPVVE	43	55	1.52
			sSDNGTTSPVVE	44	55	1.29
AT5G51545.1	Low PSII accumulation2	LPA2	qNSQIESDTTEDPSR	32	46	1.70
AT5G53460.1	NADH‐dependent glutamate synthase 1	GLT1	cGVGFVAE	117	124	1.02
AT5G58290.1	Regulatory particle triple‐A ATPase 3	RPT3	aSAAVASmVLDPkASPALMD	Ac‐2	21	2.12

Peptides listed are more than two‐fold increased in abundance in *prt6‐5*, compared to Col‐0, at *P *<* *0.05. The start and finish amino acid positions are defined with respect to TAIR10 gene models. Residues with modifications Nt‐TMT, side‐chain Lys TMT or other (e.g. oxidised Met) are indicated in lower case; full details are given in Supporting Information Table S3. Ac, N‐terminal acetylation.

### Increased abundance of seed storage proteins in *prt6* seedlings requires RAP‐type ERFVII transcription factors

**Figure 4:**
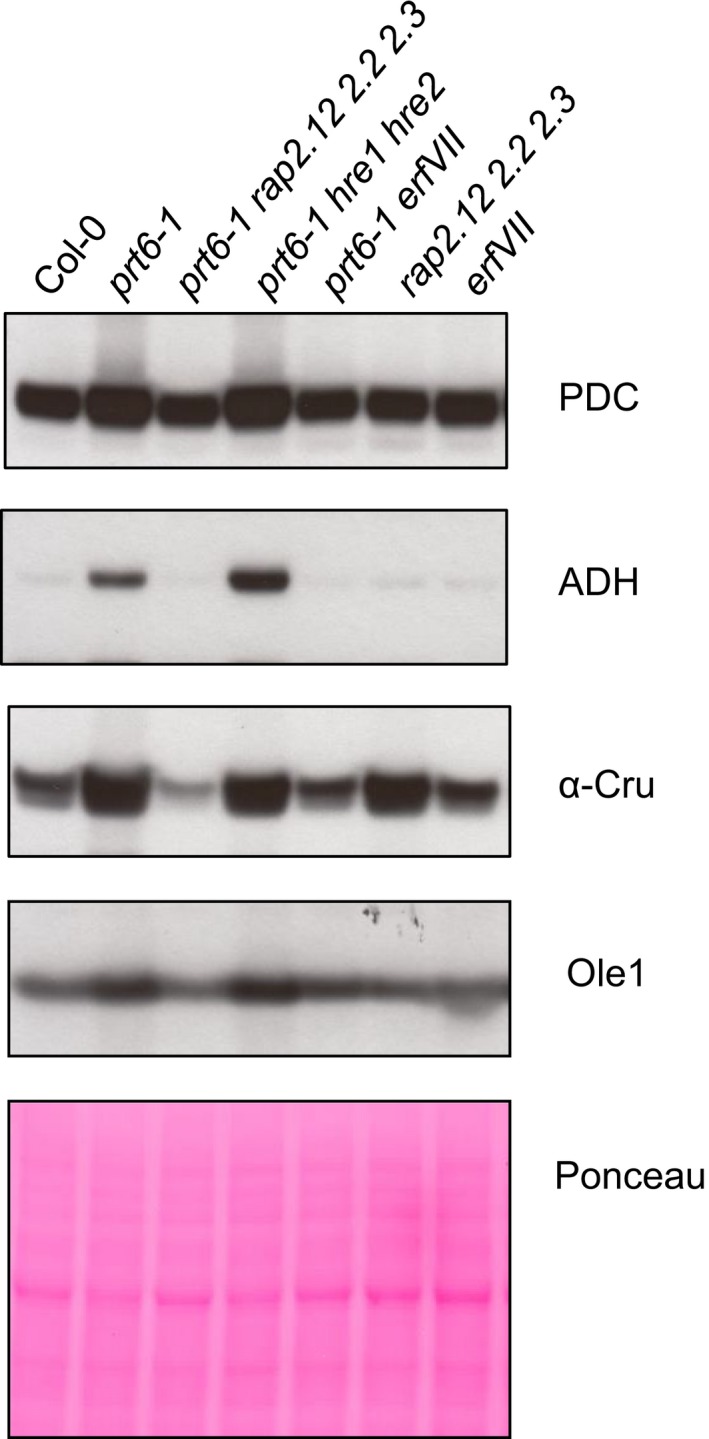
Increased abundance of proteins in *prt6* seedlings requires RAP‐type ERFVII transcription factors. Proteins were extracted from 4‐d‐old etiolated seedlings of *Arabidopsis thaliana* N‐end rule and *erfVII* combination mutants and subjected to immunoblotting (25 µg per lane) with antisera towards pyruvate decarboxylase (PDC), alcohol dehydrogenase (ADH), cruciferin α subunit (α‐Cru) and oleosin1 (Ole1). Representative of three independent experiments.

Cruciferins are highly abundant in embryo and endosperm of seeds but are mobilised following germination and are not normally present in 4‐d‐old seedlings. Multiple cruciferin‐derived Nt peptides were identified but only one had a primary destabilising residue. Several neo Nt peptides had secondary or tertiary destabilising residues; these did not bear the enzymatic modifications (arginylation, deamidation) required for degradation (Table S2), arguing against cruciferins being novel Arg/N‐end rule substrates and implying that cruciferin abundance is controlled directly or indirectly by stabilisation of an N‐end rule substrate in *prt6* seedlings. Since *RAP2.12*,* RAP2.2* and *RAP2.3* control the transition from dormancy to germination and seedling response to ABA (Gibbs *et al*., 2014b; Papdi *et al*., 2015), we tested whether they also underpin the role of the Arg/N‐end rule in regulating storage reserve mobilisation. Proteins extracted from 4‐d‐old seedlings of mutants lacking *PRT6* and different combinations of *ERFVIIs* were analysed by immunoblotting, using antisera towards the α‐subunit of cruciferin (Wan *et al*., 2007). Hypoxia marker proteins, ADH and pyruvate decarboxylase (PDC), both known to be regulated transcriptionally by the Arg/N‐end rule (Gibbs *et al*., 2011), were also tested and the oil body structural protein, Oleosin 1 (Ole1) was included because *prt6* exhibits an oil body retention phenotype (Holman *et al*., 2009). Signals with all four antisera were increased in both the *prt6‐1* and *prt6‐5* null mutants and the *prt6‐1 hre1 hre2* triple mutant relative to WT (Figs 4, S4). However, abundances of Ole1, ADH and PDC in quadruple *prt6‐1 rap2.12 rap2.2 rap2.3* and sextuple *prt6‐1 rap2.12 rap2.2 rap2.3 hre1 hre2* (hereafter, ‘*prt6 erf VII* ’) mutant seedlings were comparable to those in WT. Abundance of α‐cruciferin in the sextuple mutant was also similar to that in Col‐0, but was reproducibly lower in the quadruple mutant, suggesting possible feedback regulation by HRE1 and/or HRE2 (Figs 4, S4). The *rap2.12 rap2.2 rap2.3* triple mutant had a surprisingly high level of α‐cruciferin but all proteins (including α‐cruciferin) were present at wild type amounts in plants lacking all five ERFVII transcription factors (*erf VII*). Taken together, removal of *PRT6* function is associated with increased abundance of the storage protein cruciferin, which can be attributed to the action of *PRT6* on different members of the ERFVII transcription factor family.

### Mobilisation of cruciferin is aberrant and delayed in Arg/N‐end rule mutants

**Figure 5:**
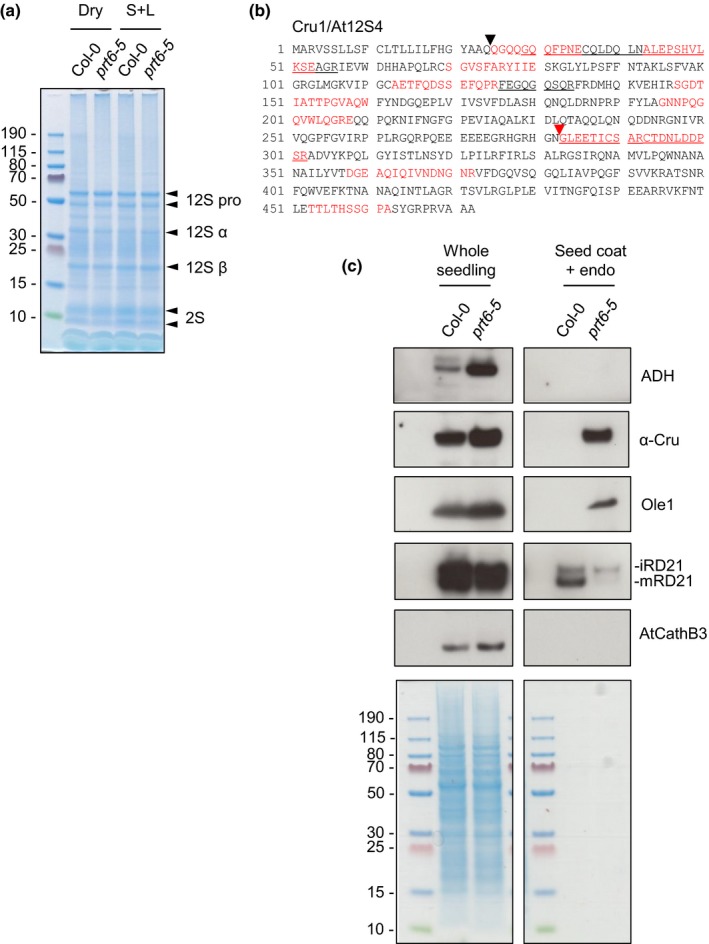
Mobilisation of 12S seed storage proteins is impaired in *prt6* endosperm. (a) Quick Coomassie blue‐stained gel of proteins extracted from dry and germinating *Arabidopsis thaliana* seeds (protein extracted from five seeds was loaded in each lane). Dry, dry seed; S + L, seeds after 48 h of stratification on agar plus 6 h of light. The arrows indicate positions of the 12S cruciferin pro‐protein (pro), α and β subunits, and 2S albumins (napins). (b) Amino acid sequence of CRU1/At12S4; peptides identified by TMT™‐TAILS are indicated in red and/or underlined (some peptide sequences overlap). Black arrow indicates Nt of the α‐subunit generated by removal of residues 1–24; red arrow indicates Nt of the β‐subunit generated by proteolytic processing. (c) Immunoblots of 4‐d‐old seedlings, with endosperm and seed coat attached (loading equivalent to eight seedlings) and 15 dissected endosperms plus seed coat, probed with antisera towards alcohol dehydrogenase (ADH), cruciferin α subunit (α‐Cru), oleosin 1 (Ole1), RD21A (identifies both intermediate and mature forms, iRD21 and mRD21, respectively) and AtCathB3. The panel below shows the corresponding Quick Commassie blue‐stained gel; positions of molecular weight markers (kDa) are indicated to the left of the panel.

Examination of the protein profiles of dry and imbibed seeds indicated that *prt6‐5* and wild type seeds contain similar amounts of storage proteins (Fig. 5a), and therefore the difference in cruciferin abundance between the two genotypes is established following germination. The polypeptide pattern of *prt6‐5* seeds was consistent with correct processing of seed storage proteins during maturation. In agreement with this, neo‐Nt peptides corresponding to the α‐ and β‐subunit N‐termini of Cru1/At12S4 and Cru3/At12S1 (as defined by Higashi *et al*., 2006) were identified in 4‐d‐old etiolated seedlings, as were peptides corresponding to the β‐subunit N‐termini of Cru2/At12S3 and At12S2 (Figs 5b, S5). During germination, the α‐subunits of cruciferins are degraded successively from the C‐terminus (Higashi *et al*., 2006; Li *et al*., 2007). However, numerous different neo‐Nt peptides derived from the α‐subunits of the four cruciferins were observed in the N‐terminome dataset, indicative of aberrant degradation (Figs 5b, S5). To determine whether the presence of seed storage proteins detected in *prt6* seedlings was a result of delayed mobilisation, seed coat and endosperm were dissected from 4‐d‐old etiolated seedlings and analysed separately by immunoblotting. ADH, α‐cruciferin and Ole1 exhibited increased abundance in intact *prt6‐5* seedlings. Whilst endosperms of germinated *prt6‐5* seeds contained α‐cruciferin and Ole1, these proteins were not detected in wild type endosperm (Fig. 5c). ADH was strongly upregulated in whole *prt6‐5* seedlings but absent from seed coat and endosperm in both mutant and wild type. These findings demonstrated that removal of *PRT6* function inhibits seed reserve mobilisation.

### Multiple protein groups exhibit reduced abundance in the N‐terminome of *prt6* seedlings

Peptides representing 101 protein groups were significantly reduced in abundance in *prt6* seedlings relative to Col‐0 (Table 2). As analysis of gene ontogeny terms was uninformative, proteins were categorised manually. N‐termini of various proteins associated with the apoplast and cell wall were downregulated in *prt6*, including xylan‐modifying enzymes, β‐galactosidases and a prolyl 4‐hydroxylase that modifies extensin proteins. Numerous chloroplast proteins were also represented in the *prt6* downregulated dataset, most notably proteins involved in Chl biosynthesis, consistent with the known role for the Arg/N‐end rule pathway in regulating tetrapyrrole synthesis as part of photomorphogenesis (Abbas *et al*., 2015). Other proteins with reduced abundance in the *prt6* N‐terminome included a disparate group of enzymes involved in carbon metabolism and, interestingly, all enzymes of the *S*‐adenosyl methionine (SAM) cycle. Finally, N‐terminal peptides of seven proteases were decreased in abundance in *prt6*.

**Table 2:** N‐terminal peptides with decreased abundance in seedlings of the *Arabidopsis thaliana prt6* mutant

AGI code	Description	Synonyms	Peptide	Start	Finish	Log_2_ fold change
Proteases and inhibitors						
AT1G47128.1	Granulin repeat cysteine protease family protein	RD21A	dELPESIDWR	135	144	−1.02
AT3G14067.1	Subtilase family protein	SASP	sAGNSGPNPE	318	327	−1.05
			aGNSGPNPE	319	327	−1.01
AT3G45010.1	Serine carboxypeptidase‐like 48	Scp148	gSGGSPSVQDFGH	89	101	−1.47
AT4G34980.1	Subtilisin‐like serine protease 2	SLP2	aVGSNEGDR	444	452	−1.20
AT4G36195.1	Serine carboxypeptidase S28 family protein		tAVTPESADR	327	336	−1.56
AT4G36880.1	Cysteine proteinase1	CP1, RDL1	gkEVPETVDWR	142	152	−1.30
AT4G39090.1	Papain family cysteine protease	RD19A	aAGYAPAR	311	318	−1.01
AT3G12490.2	Cystatin B	CYSB, AtCYS6	dVPANQNSGEVESLAR	42	57	−1.84
C metabolism						
AT1G32710.1	Cytochrome *c* oxidase, subunit Vib family protein		sSAQMDPHDkMR	Ac‐2	13	−1.03
AT1G53310.1	Phosphoenolpyruvate carboxylase 1	PPC1	nLAEEVQIAYR	106	116	−1.29
AT2G30970.1	Aspartate aminotransferase 1	ASP1	sTILEDPE	326	333	−1.42
AT3G23940.1	Dihydroxy acid dehydratase	DHAD	mTVTGQTLAQNLE	392	404	−1.44
AT3G55410.1	2‐Oxoglutarate dehydrogenase, E1 component		gEVSQQDIDR	536	545	−1.09
AT5G14740.1	Carbonic anhydrase 2	CA2	sFPLDGNNSTDFIE	233	246	−1.51
AT5G39410.1	Saccharopine dehydrogenase		gFDSIPAE	153	160	−1.40
Lipid metabolism						
AT1G30120.1	Pyruvate dehydrogenase E1 beta		lSSQDVPTPYAGTLE	373	387	−1.01
AT1G55020.1	Lipoxygenase 1	LOX1	tLEDVPGHGR	129	138	−1.01
AT3G06650.1	ATP‐citrate lyase B‐1	ACLB‐1	vSGAHNTIVTAR	417	428	−1.55
AT3G16170.1	AMP‐dependent synthetase and ligase family protein	AAE13	nQFQDDSFE	155	163	−1.75
AT5G43590.1	Acyl transferase/acyl hydrolase/lysophospholipase superfamily protein		sLDGGGVR	13	20	−1.23
AT5G46290.3	3‐Ketoacyl‐acyl carrier protein synthase I	KAS1	dVDAYYE	77	83	−1.04
One‐carbon metabolism and ethylene						
AT1G05010.1	Ethylene‐forming enzyme	ACCO, ACO4	mESFPIINLEkLNGEER	Ac‐1	17	−1.50
AT3G09820.1	Adenosine kinase 1	AK1	lPYmDYIFGNE	213	223	−1.09
AT3G59970.3	Methylenetetrahydrofolate reductase 1	MTHFR	aLDLVNHIR	131	139	−1.59
AT4G01850.1	*S*‐Adenosylmethionine synthetase 2	SAM‐2	gTGLIPDkE	324	332	−1.06
AT4G13940.1	*S*‐Adenosyl‐l‐homocysteine hydrolase	HOG1	sFTNQVIAQLE	412	422	−1.75
AT5G14780.1	Formate dehydrogenase	FDH	vENALGIR	59	66	−1.33
AT5G17920.1	Methioine synthase	ATMS1	lQAFTGAYAE	219	228	−1.49
			aGIGPGVYDIHSPR	691	704	−1.09
Photosynthesis and Chl biosynthesis						
AT4G27440.1	Protochlorophyllide oxidoreductase B	PORB	akEPTYSAE	181	189	−1.78
			aLFPPFQkY	325	333	−1.72
			iASTGLFRE	310	318	−1.40
			nAAVYFPTAkEPTYSAE	173	189	−1.30
			iASTGLFR	310	317	−1.16
AT5G08280.1	Hydroxymethylbilane synthase	HEMC, RUGOSA	sLNHEETR	292	299	−1.24
AT5G54190.1	Protochlorophyllide oxidoreductase A	PORA	aIATSTPSVTkSSLDR	Ac‐71	86	−1.19
			nAAVYQPTANQPTFTAE	177	193	−1.09
AT5G64040.2	Photosystem I reaction centre subunit PSI‐N	PSAN	gVIDEYLER	87	95	−1.45
ATCG00120.1	ATP synthase subunit alpha	ATPA	qSQSAPLTVEE	423	433	−1.46
ATCG00480.1	ATP synthase subunit beta	PB	iVGEEHYETAQQVkQ	379	393	−1.04
ATCG00490.1	Ribulose‐bisphosphate carboxylases	RBCL	dDYVEkDR	351	358	−1.50
Other plastid						
AT1G12230.2	Aldolase superfamily protein		nEIDVPHDR	211	219	−1.30
AT1G34000.1	One‐helix protein 2	OHP2	sQTEGPLR	44	51	−1.47
AT2G21530.1	SMAD/FHA domain‐containing protein		lDENQSPTSGGER	74	86	−1.09
AT2G23670.1	Homologue of Synechocystis YCF37	YCF37	eNIPLFGIR	72	80	−1.11
AT2G44920.2	Tetratricopeptide repeat (TPR)‐like superfamily protein		aSFFDADLTGADLSEADLR	131	149	−2.47
AT3G56910.1	Plastid‐specific 50S ribosomal protein 5	PSRP5	kAAASGVDGAEPE	Ac‐64	76	−1.89
			aAASGVDGAEPE	65	76	−1.82
			sGVDGAEPE	68	76	−1.73
AT4G32915.1	Glu‐tRNA Gln amidotransferase, C subunit		sSDSDSSVLQPPDVAR	53	68	−1.83
AT4G34290.1	SWIB/MDM2 domain superfamily protein		aASSDPTTTTkTR	Ac‐51	63	−1.17
AT5G02710.1	Unknown protein		sTSGFSGGTTkE	Ac‐43	54	−1.09
Apoplast/cell wall						
AT1G68560.1	Alpha‐xylosidase 1	XYL1	dEEENkSVMVEVR	884	896	−1.08
AT2G05380.1	Glycine‐rich protein 3 short isoform	GRP3S	gGGFGDNGGGR	41	51	−1.03
AT2G17720.1	2‐Oxoglutarate (2OG) and Fe(II)‐dependent oxygenase superfamily protein	P4H5	dVDDGGETVFPAAR	207	220	−2.10
AT2G39770.1	Glucose‐1‐phosphate adenylyltransferase family protein	VTC1, CYT1	sTVGQWAR	313	320	−1.48
AT3G13790.1	Glycosyl hydrolases family 32 protein	ATBFRUCT1	sPSVNQPYR	44	52	−1.78
AT3G44990.1	Xyloglucan endo‐transglycosylase‐related 8	XTH31	fFVDDVPIR	162	170	−1.60
AT4G14130.1	Xyloglucan endotransglucosylase/hydrolase 15	XTH15	yLSSQGATHDE	92	102	−1.48
AT4G32460.1	Protein of unknown function, DUF642		gPLIDGVAmR	172	181	−1.09
AT5G20630.1	Germin 3	GER3	kNPDQVTE	42	49	−1.45
AT5G20710.1	Beta‐galactosidase 7	BGAL7	tIVSHDER	26	33	−1.02
AT5G44380.1	FAD‐binding Berberine family protein		sASIQDQFINcVkR	31	44	−1.26
AT5G46960.1	Plant invertase/pectin methylesterase inhibitor superfamily protein		vNSLTQDPQSkAATTLE	44	60	−2.13
AT5G56870.1	Beta‐galactosidase 4	BGAL4	dITIGSGE	475	482	−1.04
AT5G64100.1	Peroxidase superfamily protein		sIPANAPGILR	63	73	−1.04
Cytoskeleton						
AT1G71440.1	Tubulin folding cofactor E/Pfifferling (PFI)	PFI	mkAESSNESFIIGQR	Ac‐1	15	−1.14
AT3G60830.1	Actin‐related protein 7	ARP7	nVSGFYASE	116	124	−1.15
AT5G55230.2	Microtubule‐associated proteins 65‐1	MAP65‐1	aVTDTESPHLGE	2	13	−1.42
Vesicle traffic and organelle biogenesis						
AT1G35720.1	Annexin 1	ANNAT1	dSVPAPSDDAE	8	18	−1.07
AT1G71820.2	SEC6	SEC6	mMVEDLGVEAkEAAVR	Ac1	16	−1.23
AT4G11380.2	Adaptin family protein		aLFGEDGR	802	809	−1.16
Chaperones						
AT1G24510.1	TCP‐1/cpn60 chaperonin family protein		nDVGTNDmR	490	498	−1.81
AT2G33210.1	Heat shock protein 60‐2	HSP60‐2	sVSSLLTTTE	541	550	−1.09
AT3G12050.1	Aha1 domain‐containing protein		gLVDMPYISDE	108	118	−1.20
AT3G44110.1	DNAJ homologue 3	J3	eETTLHDVNIEDEmR	375	389	−1.22
AT4G24190.1	Chaperone protein htpG family protein	SHD	iSPDAVADEE	772	781	−1.33
			tDSDVVHR	55	62	−1.02
AT5G53400.1	HSP20‐like chaperones superfamily protein	BOB1	aSSAEPIE	111	118	−1.05
AT5G56030.2	Heat shock protein 81‐2	HSP81‐2	gLSIDDDDAVE	695	705	−2.63
			iDDDDAVE	698	705	−2.26
			lSIDDDDAVE	696	705	−1.95
Translation						
AT2G20450.1	Ribosomal protein L14		sLTDIVIDINR	54	64	−1.17
			dVVDQNR	29	35	−1.14
AT2G27710.1	60S acidic ribosomal protein family		vASATSGGGGGGGASAAE	75	92	−1.02
AT5G47880.1	Eukaryotic release factor 1‐1		gLVLYTGTIVNE	91	102	−1.67
Nucleic acid binding						
AT1G22300.1	General regulatory factor 10	GCRF10	dLNEEGDER	235	243	−2.80
			gLAPTHPVR	163	171	−2.09
AT2G14285.1	Small nuclear ribonucleoprotein family protein		nVLYVRGVPE	42	51	−2.78
AT2G35410.1	RNA‐binding (RRM/RBD/RNP motifs) family protein		aADFNPVSAR	216	225	−1.16
AT3G59980.1	Nucleic acid‐binding, OB‐fold‐like protein		aAPDAGTTVSADE	76	88	−1.78
AT5G47210.1	Hyaluronan/mRNA binding family		dDAEDPSQLAVALSQkVE	12	29	−1.58
Redox/stress						
AT3G01520.1	Adenine nucleotide alpha hydrolases‐like superfamily protein		vVDEDGFDDVDSIYASPEDFR	55	75	−1.29
AT4G11600.1	Glutathione peroxidase 6	GPX6	vASQcGLTNSNYTE	101	114	−1.42
AT5G54430.1	Adenine nucleotide alpha hydrolases‐like superfamily protein	PHOS32	tQIEDPNAQPQPSQE	101	115	−1.34
Other						
AT1G77540.1	Acyl‐CoA N‐acyltransferases (NAT) superfamily protein		tNTAATTEAkMATEPPkIVW	Ac‐2	21	−2.06
AT2G01530.1	MLP‐like protein 329	MLP329	aTSGTYVTEVPLkGSADkH	Ac‐2	20	−1.10
AT2G26210.1	Ankyrin repeat family protein		aGLDTPQR	90	97	−1.16
AT2G38710.1	AMMECR1 family		tVSVLTDYE	96	104	−1.24
AT2G39310.1	Jacalin‐related lectin 22	JAL22	gGEGGQEWDDDVYEGVR	12	28	−1.88
AT2G44060.1	Late embryogenesis abundant protein, group 2		kEDDDDDDEE	316	325	−1.07
AT3G02090.2	Insulinase (Peptidase family M16) protein	MPPBETA	gTSPIAEDIGR	456	466	−1.41
AT3G43810.1	Calmodulin 7	CAM7	aDQLTDDQISEFkEAF	Ac‐2	17	−1.61
AT4G23400.1	Plasma membrane intrinsic protein 1;5	PIP1;5	mEGkEEDVNVGAN	Ac‐1	13	−1.03
AT4G24520.1	P450 reductase 1	ATR1	vATYGDGEPTDNAAR	145	159	−1.56
AT5G11950.1	Putative lysine decarboxylase family protein	LOG8	dTGVEEGFIkPGAR	155	168	−1.66
AT5G16280.1	Tetratricopeptide repeat (TPR)‐like superfamily protein		aLTGDDIVE	258	266	−1.38
AT5G44020.1	HAD superfamily, subfamily IIIB acid phosphatase		sSQYEDDVER	88	97	−1.26
Unknown						
AT2G23370.1	Unknown protein		sLEGTWDESLER	308	319	−1.04
AT2G32240.1	FUNCTIONS IN: molecular function unknown; INVOLVED IN: response to cadmium ion		dIDLSFSSPTkR	1267	1278	−1.62
AT2G38450.1	CONTAINS InterPro DOMAIN/s: Sel1‐like (InterPro:IPR006597)		mDSSDkDSSSTTTTSETTR	Ac‐34	52	−1.87
AT3G03150.1	Unknown protein		gHSSAYDkNVE	40	50	−1.09
AT5G40450.1	Unknown protein		eSSDEALVSm	1897	1906	−1.14
AT5G67490.1	Unknown protein		sSGTPPPPQAPSPNQDLNR	29	47	−1.29

Peptides listed are more than two‐fold decreased in abundance in *prt6*, compared to Col‐0, at *P *<* *0.05. The start and finish amino acid positions are defined with respect to TAIR10 gene models. Residues with modifications Nt‐TMT, side‐chain Lys TMT or other (e.g. oxidised Met) are indicated in lower case; full details are given in Supporting Information Table S3. Ac, N‐terminal acetylation.

### Proteases are differentially regulated in *prt6*

**Figure 6:**
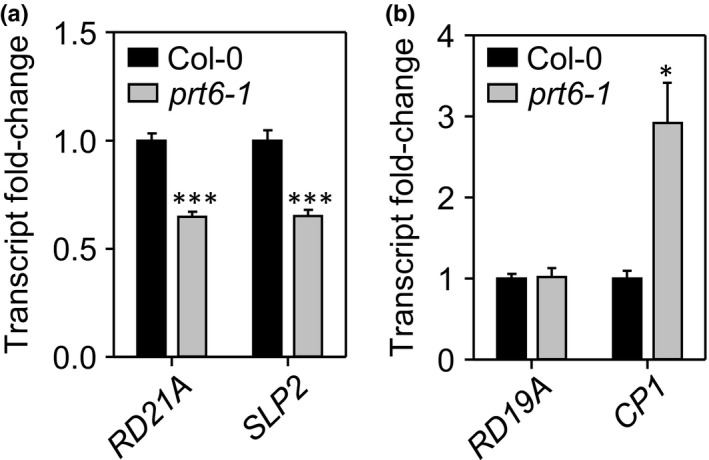
Quantification of protease transcripts in Col‐0 and *prt6* seedlings. RT‐qPCR analysis of (a) *RD21A* and *SLP2*, and (b) *RD19A* and *CP1* in 4‐d‐old etiolated *Arabidopsis thaliana* seedlings of Col‐0 and *prt6‐1*. Values are means ± SE (*n *=* *4); *, *P *<* *0.05; ***, *P *<* *0.001.

**Figure 7:**
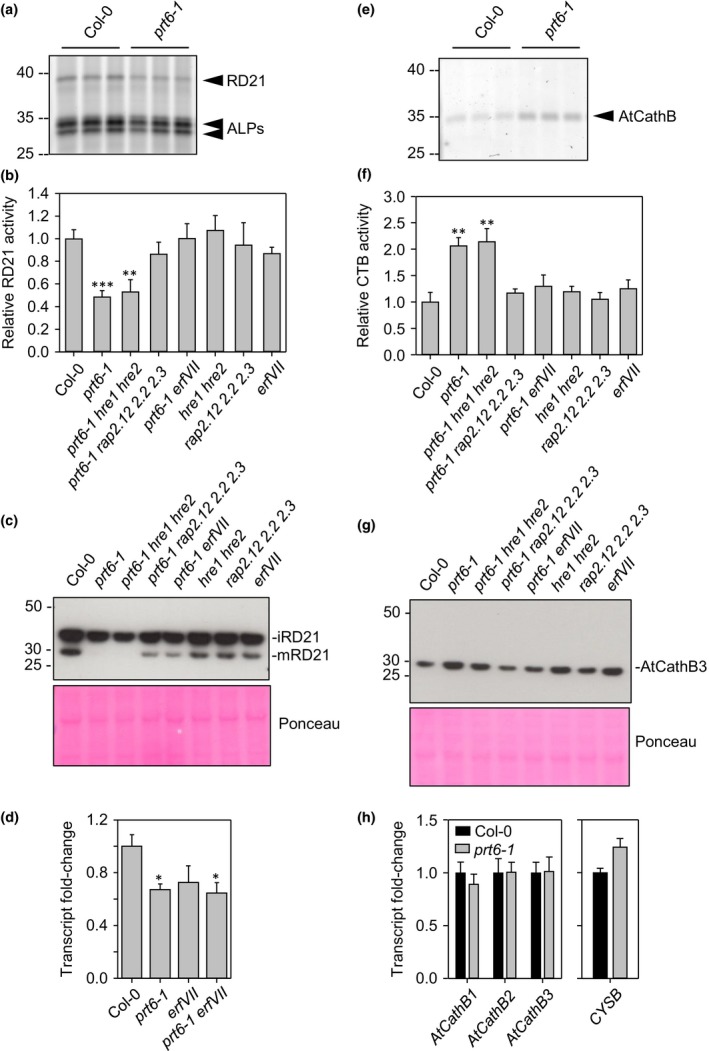
Differential regulation of proteases by the Arg/N‐end rule pathway. Activities, protein and RT‐qPCR analysis of selected *Arabidopsis thaliana* proteases. (a, e) Activity‐based protein profiling of 4‐d‐old etiolated seedlings of N‐end rule and *erfVII* combination mutants. (a) Probe FY01 labels RD21A and aleurin‐like proteases (ALPs); (e) probe JOGDA1 labels cathepsin B (AtCathB). Each lane represents a biological replicate; positions of molecular weight markers (kDa) are shown to the left of each panel. (b, f) Quantification of (b) RD21A signal and (f) AtCathB signal; values are means ± SE (*n *=* *3). (c, g) Immunoblots probed with antisera raised to (c) RD21A and (g) AtCathB3; protein extracts from equal numbers of 4‐d‐old etiolated seedlings were loaded in each lane; positions of molecular weight markers (kDa) are shown to the left of each panel. (d, h) RT‐qPCR analysis of (d) *RD21A*, and (h) *AtCathB1‐3* and *CYSB* in etiolated seedlings of Col‐0 and *prt6‐1*. Values are means ± SE (*n *=* *4); *, *P *<* *0.05; **, *P *<* *0.01; ***, *P *<* *0.001.

The reduced abundance of protease N‐termini in *prt6* was of interest in the context of delayed seed storage protein mobilisation. To gain insight into their potential regulation by the Arg/N‐end rule, transcript levels of selected proteases were quantified by RT‐qPCR. *RD21A* and *SLP2* transcripts were less abundant in *prt6‐1* seedlings than Col‐0; *RD19A* was unchanged and *CP1* was more abundant in *prt6‐1*, indicative of distinct modes of control (Fig. 6). As it is generally not possible to predict protease activity from transcript or even protein abundance (van der Hoorn, 2008), we took advantage of the availability of fluorescent probes for ABPP of cysteine proteases to examine a potential role of the Arg/N‐end rule in protease regulation (Richau *et al*., 2012; Lu *et al*., 2015). Specificity of the probes has been established previously by analysis of Arabidopsis protease knock‐out lines and transient expression of proteases in *Nicotiana benthamiana* (Gu *et al*., 2012; Lu *et al*., 2015); labelling specificity was confirmed here by pre‐incubation with the inhibitor, E64 (Fig. S6). Figure 7 shows ABPP results for FY01 and JODGA1 probes; images of the whole gels are shown in Fig. S7. Dependent on the labelling conditions, FY01 detects aleurain‐like proteases (ALPs) and RD21A (Lu *et al*., 2015). In extracts of 4‐d‐old etiolated seedlings, FY01 labelled bands of 30 and 34 kDa, probably corresponding to ALPs, AALP and ALP2, respectively, and a band of *c*. 40 kDa, corresponding to iRD21A, the active intermediate form of RD21A (Fig. 7a; note that labelling alters the apparent relative molecular mass of the proteases). Intensity of the RD21A signal was reduced in *prt6‐1* and the *prt6‐1 hre1 hre2* triple mutant, but not significantly different from WT in the *prt6‐1 rap2.12 rap2.2 rap2.3* quadruple mutant and the *prt6 erf VII* sextuple mutant, indicating that repression of RD21A activity is dependent on RAP transcription factors (Fig. 7b). Consistent with the implication of RAPs, RD21A was also downregulated in *ate1 ate2*, which lacks arginyl transferase function (Fig. S6). MV201, which also labels RD21A and other papain‐like cysteine proteases (PLCPs) (Richau *et al*., 2012), gave a similar result (Figs S6c, S7).

RD21A undergoes several processing steps: the signal peptide is cleaved co‐translationally and removal of the inhibitory pro‐domain generates an active, intermediate form (iRD21). The protein is further matured by removal of the C‐terminal granulin domain to produce two additional activated forms (mRD21) (Yamada *et al*., 2001; Gu *et al*., 2012). Two overlapping Nt peptides (DELPESIDWR; ELPESIDWR) corresponding to the N‐terminus of the activated form (iRD21) and indicative of ‘ragged’ processing were of significantly lower abundance in *prt6‐5* (two‐ and 1.75‐fold lower than Col‐0, respectively; *P *<* *0.05). RD21A protein abundance and processing were investigated further using an antiserum raised to the N‐terminus of the active, processed form (Kaschani *et al*., 2009). Bands corresponding to the intermediate form (iRD21) and the mature forms (mRD21) were detected in Col‐0, but the iRD21 band was less intense in *prt6‐1* and mRD21 was undetectable (Figs 7c, S8). Combining *hre1* and *hre2* alleles with *prt6‐1* did not recapitulate the WT phenotype, indicating that HRE1 and HRE2 do not play a role in downregulation of RD21 in the *prt6* background. By contrast, removal of ERFVII function in either the sextuple *prt6 erf VII* mutant or the quadruple *prt6 rap2.12 rap 2.2 rap 2.3* increased the abundance of iRD21 and mRD21 (Figs 7c, S8). Given the RAP‐dependence of RD21A activity and protein, we quantified transcripts in different genetic backgrounds. *RD21A* transcript levels were reduced not only in *prt6‐1* seedlings (as in Fig. 6) but also in *erf VII* and *prt6 erf VII* mutants (Fig. 7d).

JODGA1 labelled a band of *c*. 34 kDa, which corresponds to the cathepsin B (AtCathB)‐specific signal detected with this probe (Lu *et al*., 2015). Intriguingly, this signal was significantly increased in *prt6‐1*,* prt6‐5* and *ate1 ate2*, indicating an enhancement of cathepsin activity (Figs 7e, S6d). Probing extracts from combination mutants impaired in function of different ERFVII transcription factors demonstrated that this effect was dependent on RAP transcription factors but independent of HRE1 and HRE2 (Figs 7f, S7). Although no cathepsin‐derived peptides were identified in the N‐terminome dataset, immunoblotting with a specific antiserum confirmed that AtCathB3 exhibited increased abundance in *prt6‐1* and the *prt6‐1 hre1 hre2* triple mutant (Fig. 7g). Removing RAP function in the quadruple *prt6‐1 rap2.12 rap 2.2 rap 2.3* or the sextuple *prt6 erf VII* mutants restored AtCathB3 protein to WT levels but the interpretation of this result was complicated by higher levels of AtCathB3 in the *erf VII* pentuple and lower‐order mutants (which are wild type for *PRT6*). Despite the apparent *RAP*‐dependence of increased AtCathB3 protein and activity in *prt6* seedlings, cathepsin B transcripts were not increased in the mutant, pointing to post‐transcriptional or post‐translational regulation (Fig. 7h). Therefore, we tested whether altered expression of the seed‐expressed cystatin, AtCYS6/CYSB (At3g12490; Hwang *et al*., 2009), might contribute to post‐translational regulation of AtCathB3 in *prt6‐1*. Cystatins are candidate regulators of cathepsin activity and a CYSB‐derived peptide was present in the *prt6*‐down dataset (Table 2), but other peptides from this protein were not changed in abundance in the mutant (Table S2) and *CYSB* transcripts were not reduced in *prt6‐1* relative to WT (Fig. 7h). Taken together, the ABPP, immunoblotting and transcript data indicate regulation of proteases by the Arg/N‐end rule at both transcriptional and post‐transcriptional levels, which is largely, but not completely, RAP‐dependent.

## Discussion

### Impact of the Arg/N‐end rule on the proteome

In recent years, the N‐end rule pathway of targeted protein degradation has emerged as an important regulator of diverse processes in plants (Gibbs *et al*., 2014b, 2015, 2016). Whilst analysis of mutants impaired in different pathway components has provided insight into the physiological functions of the N‐end rule, knowledge regarding the identity of substrates and the impact of this pathway on the proteome is limited. Affinity purification and quantitative proteomics provide unbiased strategies to probe the N‐end rule in plants. Previously, we used TMT labelling and tandem MS to identify proteins with altered abundance in roots of *prt6* and *ate1 ate2* mutants, and employed dimethyl‐TAILS to isolate Nt peptides, which achieved high enrichment of protein N‐termini but did not allow reliable quantification (Zhang *et al*., 2015). Here, we combined the TAILS technique with TMT labelling for enrichment of Nt peptides in etiolated *prt6* seedlings. The TMT‐TAILS protocol enabled robust quantification, with quantitative data obtained for 3937 peptides (Table S2; Fig. S3). Moreover, the use of two proteases increased coverage and increased the proportion of neo‐Nt peptides identified (Fig. 2b). This dataset provides a useful resource for proteogenomics: although not a focus of our study, the data can be used, for example, to identify proteolytic processing events, such as those involved in protein in/activation and signal peptide removal and to support analysis of alternative translation start sites (Hartmann & Armengaud, 2014).

Regarding the Arg/N‐end rule, neo‐Nt peptides with destabilising residues were under‐represented in the complete dataset (Fig. 2g), but were not markedly upregulated in *prt6* compared to wild type (Table S3). Consistent with our results from global TMT labelling (Zhang *et al*., 2015), relatively few proteins exhibited altered abundance in *prt6*, implying that the *PRT6* does not function in bulk protein turnover under normal conditions, but probably plays a role in the controlled degradation of a few regulatory proteins. In agreement with this, it is clear from global protein lifetime measurements and several N‐terminome studies that by no means all proteins with destabilising N‐termini are degraded via the N‐end rule in wild type plants (Bienvenut *et al*., 2012; Tsiatsiani *et al*., 2013; Linster *et al*., 2015; Venne *et al*., 2015; Zhang *et al*., 2015; Li *et al*., 2017). Degradation depends on the structural and subcellular context of the destabilising residue (amongst other factors) as part of a functional N‐degron (Varshavsky, 2011).

### Upregulation of proteins in *prt6* seedlings requires ERFVII transcription factors

Of the 45 protein groups upregulated in *prt6‐5* relative to Col‐0, there were no obvious candidate Arg/N‐end rule substrates nor were the known ERFVII substrates identified, suggesting that further enrichment is required to detect low abundance, regulatory proteins. The abundance of cruciferin was dependent on activity of RAP‐type ERFVIIs, indicating that cruciferin is controlled by the known Arg/N‐end rule substrates, rather than being a substrate itself (Fig. 4). Immunoblotting confirmed the ERFVII‐dependent up‐regulation of ADH and PDC, in agreement with their known roles in the low oxygen response (Licausi *et al*., 2013; Gibbs *et al*., 2015), and demonstrated that Oleosin1 is regulated by RAP‐type ERFVIIs (Fig. 4). Finally, although not identified previously as *prt6*‐regulated in published transcriptome datasets, we demonstrated a RAP‐dependent increase of *PYL2* transcripts in *prt6* seedlings which was reflected in protein abundance; upregulation of this ABA receptor component may contribute to the ABA hypersensitivity of *prt6* (Holman *et al*., 2009).

### Protein groups associated with diverse functions are downregulated in *prt6*

Nt peptides representing a diverse collection of proteins were downregulated in *prt6* seedlings (Table 2). Whilst these data require confirmation at the protein level with immunoblotting or quantitative shotgun proteomics, they nevertheless support the notion that stabilisation of Arg/N‐end rule substrates can impact negatively on abundance of other proteins, either directly or indirectly. Numerous transcripts are downregulated in published microarray data from different tissues of Arg/N‐end rule mutants (Choy *et al*., 2008; Gibbs *et al*., 2011; de Marchi *et al*., 2016). This suggests that transcriptional repression by stabilised ERFVIIs or unknown transcription factor substrates probably underpins the down‐regulation of protein abundance in *prt6*, although other mechanisms are also possible. N‐termini of several plastid proteins, including enzymes of Chl biosynthesis, were downregulated in *prt6* seedlings, consistent with the known role for the Arg/N‐end rule pathway in co‐ordination of photomorphogenesis and oxygen sensing (Abbas *et al*., 2015). Transcripts of nuclear photosynthesis‐related genes are markedly lower in dark‐grown seedlings of the *prt6* allele, *ged1*, than in wild type (Choy *et al*., 2008) and *ERF*‐dependent repression of *protochlorophyllide reductase A*,* B* and *C* and other Chl biosynthetic genes may serve to prevent accumulation of toxic metabolites under low oxygen, which is needed for several steps of Chl biosynthesis (Abbas *et al*., 2015). Our data demonstrate that these transcriptional changes are reflected at the protein level. Enzymes associated with SAM synthesis and recycling were also downregulated in the *prt6* N‐terminome. These included the three SAM cycle enzymes, Met synthase, SAM synthase and *S*‐adenosyl‐L‐homocysteine hydrolase (Table 2). Also downregulated were methylenetetrahydrofolate reductase 1 which can serve as a methyl donor for Met synthesis, and adenosine kinase, involved in salvage of adenylates and methyl recycling. SAM is an abundant cofactor required for ethylene and polyamine biosynthesis and is an important methyl donor for numerous methyltransferase reactions (Sauter *et al*., 2013), so modulation of the SAM cycle by the Arg/N‐end rule could potentially have several important metabolic and developmental consequences. Whilst the ethylene biosynthetic protein ACC oxidase4 was downregulated in the *prt6* N‐terminome, ACC oxidase1 was significantly upregulated (Table 1) and is a core hypoxia‐responsive gene constitutively expressed in *prt6* alleles (Mustroph *et al*., 2009; Gibbs *et al*., 2011). In future studies, it will be interesting to measure SAM, polyamines and ethylene in *prt6* seedlings and to determine whether ERFVII transcription factors are involved in their regulation.

### The Arg/N‐end rule differentially regulates protease activities

The N‐termini of several proteases were downregulated in the *prt6* N‐terminome, two of which, RD21A and SLP2, were also downregulated at the transcript level (Fig. 6). However, as proteases are subject to post‐translational regulation *in planta* to avoid deleterious consequences of uncontrolled proteolysis, transcript and protein levels often do not predict activity (van der Hoorn, 2008). Therefore, we analysed protease activity in *prt6* seedlings using well‐characterised, subfamily‐specific cysteine protease activity probes (Richau *et al*., 2012; Lu *et al*., 2015). Two ABPP probes, FY01 and MV201, provided evidence for reduced RD21A activity in *prt6‐1*,* prt6‐5* and *ate1 ate2* seedlings (Figs 7, S6). RD21A is responsible for the dominant PLCP activity in Arabidopsis extracts (Gu *et al*., 2012) and has been associated with functions in immunity, herbivore defence, senescence, cell death and response to stresses (Shindo *et al*., 2012; Lampl *et al*., 2013; Rustgi *et al*., 2017; and references therein). Following activation via a proteolytic cascade, RD21A activity is tightly regulated at different developmental stages by a Kunitz‐type protease inhibitor, water‐soluble Chl binding protein (reversible inhibition) and by AtSerpin1 (irreversible inhibition) (Lampl *et al*., 2013; Boex‐Fontvieille *et al*., 2015; Rustgi *et al*., 2017). RD21A protein is also subject to ubiquitin‐dependent degradation mediated by the E3 ligase AtAIRP3/LOG2 (Kim & Kim, 2013). In this study, we provide evidence for another layer of regulation via the Arg/N‐end rule pathway. RD21A activity (as quantified by ABPP) was reduced in *prt6* alleles and correlated well with protein levels assessed by MS and immunoblotting. Whilst the reduction in activity and protein was largely dependent on RAP‐type ERFVIIs, surprisingly, the transcriptional repression/downregulation of *RD21A* in *prt6* could not be clearly attributed to ERFVII function (Fig. 7).

ABPP also revealed increased Cathepsin B activity in *prt6* and *ate1 ate2* (Figs 7, S6d). Arabidopsis has three Cathepsin B genes, *AtCathB1* (At1g02300), *AtCathB2* (At1g02305) and *AtCathB3* (At4g01610), which are ubiquitously expressed (Iglesias‐Fernández *et al*., 2014) and functionally redundant in the hypersensitive response and programmed cell death (McLellan *et al*., 2009; Ge *et al*., 2016). However, *AtCathB3* exhibits the highest level of transcript, is very strongly induced in germination and accounts for the strong ABPP signal in young seedlings (Iglesias‐Fernández *et al*., 2014; Lu *et al*., 2015). Although AtCathB3 protein and activity were RAP‐dependent, surprisingly, transcript abundance was unaltered in *prt6‐1* seedlings (Fig. 7h); moreover, none of the Arabidopsis cathepsins exhibits significant differential regulation in published microarray studies of Arg/N‐end rule mutants (Choy *et al*., 2008; Gibbs *et al*., 2011). Taken together, these lines of evidence point to post‐translational regulation of AtCathB3 by the Arg/N‐end rule. Although cystatins are known regulators of Cathepsin B activity in plants, we did not find evidence for altered expression of a major seed cystatin, CYSB in *prt6* seedlings (Fig. 7h), so the RAP‐type ERFVIIs may influence AtCathB3 protein and activity via more than one cystatin or a different mechanism.

RD21A and Cathepsin B represent only a subset of the large number of proteases encoded by the Arabidopsis genome (van der Hoorn, 2008), but we have discovered that they are regulated in an opposing and complex manner by the Arg/N‐end rule. Aside from roles in seed protein mobilisation, regulation of protease activity may be important to prevent potentially deleterious accumulation of neo‐peptides in *prt6* mutants. An interesting challenge for future studies will be to determine to what extent the N‐end rule pathway regulates other proteases and to investigate the potential homeostatic interplay between different protease activities.

### The Arg/N‐end rule regulates seed storage protein mobilisation through RAP‐type ERFVII transcription factors

Identification of *PRT6* in a genetic screen for seeds with reduced germination potential provided the first link between the Arg/N‐end rule and germination completion and established a role for this pathway in storage oil mobilisation (Holman *et al*., 2009). Subsequently, we demonstrated that RAP‐type ERFVII transcription factors underpin the germination phenotype of *prt6* seeds (Gibbs *et al*., 2014a). Our quantitative proteomics data set show that the PRT6 branch of the Arg/N‐end rule pathway also plays a role in regulating breakdown of endosperm storage protein reserves (Fig. 4). In wild type plants, cruciferins are laid down during seed development and mobilised upon germination, but can also be neosynthesised following germination (Galland *et al*., 2014). Dry *prt6* and Col‐0 seeds contained similar amounts of seed storage proteins; in agreement with this, developing seeds of the *prt6* allele, *ged1* (Riber *et al*., 2015), contain wild‐type amounts of transcripts encoding seed proteins including *At12S4*/*CRU1* (Choy *et al*., 2008). Cruciferin is almost completely depleted in 4‐d‐old wild type seedlings grown in culture (Heath *et al*., 1986). The presence of cruciferin in the endosperm of *prt6* seedlings at 4 d post‐germination is suggestive of delayed mobilisation (Fig. 4). Multiple neo‐Nt peptides derived from the cruciferin α‐subunits were identified as upregulated in *prt6* seedlings, consistent with aberrant degradation and suggesting that different enzymes degrade SSPs when the protease complement of seeds is disrupted. In support of this notion, genetic removal of vacuolar processing enzymes has been shown to result in compensatory, aberrant SSP processing by alternative proteases (Gruis *et al*., 2002).

Although the reduced activity of RD21A correlates with delayed α‐cruciferin degradation in the endosperm (Fig. 5c), a causative link has not been established. Surprisingly, proteases responsible for the mobilisation of Arabidopsis SSPs have remained poorly defined until recently: whilst the activity of several proteases parallels the disappearance of cruciferins post‐imbibition, storage protein profiles were unaffected in single and multiple protease mutants (inclusive of *rd21a* alleles), indicative of substantial redundancy (Lu *et al*., 2015). Cathepsins have been associated with storage protein mobilisation in Arabidopsis (Iglesias‐Fernández *et al*., 2014), yet AtCathB3 activity and protein were increased in *prt6* seedlings, which would have been predicted to promote, not retard, SSP mobilisation. Moreover, AtCathB3 is absent from endosperm, as judged by immunoblotting (Fig. 5c) and fluorescence *in situ* hybridisation (Iglesias‐Fernández *et al*., 2014), and therefore AtCathB3 regulation by PRT6 does not affect mobilisation of SSPs in the endosperm. During the course of this study, CP1/RDL1 was shown to play a quantitatively important role in endosperm cruciferin degradation (Piskurewicz *et al*., 2016). The decay of cruciferin levels was delayed by 12 h in endosperm of *cp1* mutants, independently of germination, but embryo cruciferin was degraded normally. The SSP mobilisation phenotype of *prt6* seedlings, in which endosperm α‐cruciferin degradation is specifically retarded, is consistent with the potential regulation of CP1 by the Arg/N‐end rule. Neither an activity probe nor a specific antibody is available for CP1, so we were unable to test this hypothesis but as CP1 was represented in our *prt6* downregulated dataset (Table 2) it is plausible that it contributes to the delayed SSP mobilisation phenotype of *prt6*.

As we found previously (Zhang *et al*., 2015) many of the proteins whose abundance is influenced by the Arg/N‐end rule in this study are not *bona fide* substrates of the pathway, but are regulated (directly or indirectly) by the ERFVII transcription factors. ERFVIIs underpin many of the known *prt6* phenotypes and are emerging as the dominant substrates of PRT6 under conditions tested to date. Their role in storage reserve mobilisation during skotomorphogenesis is interesting in the context of hypoxia signalling: RAP‐type ERFVIIs play an important role in monitoring the gaseous environment during germination, which has an adaptive value in waterlogged soils and prevents precocious photomorphogenesis (Abbas *et al*., 2015). Our proteomic, biochemical and genetic data expand and complement this view, suggesting that controlled degradation of ERFVII transcription factors by the PRT6 branch of the Arg/N‐end rule pathway serves to co‐ordinate germination and seedling establishment with environmental factors by optimising storage reserve mobilisation.

## Author contributions

H.Z., K.S.L. and F.L.T. designed research, H.Z., M.J.D. and L.G. performed research, D.J.G., R.A.L.v.d.H. and M.J.H. contributed new analytical tools, H.Z., K.L.H. and F.L.T. analysed data, F.L.T. and H.Z. wrote the paper.

## Supporting information

Please note: Wiley Blackwell are not responsible for the content or functionality of any Supporting Information supplied by the authors. Any queries (other than missing material) should be directed to the *New Phytologist* Central Office.

**Fig. S1** Overview of the plant N‐end rule pathway.
**Fig. S2** The Arg/N‐end rule is active in etiolated seedlings.
**Fig. S3** Analysis of peptide quantification in Col‐0 and *prt6*.
**Fig. S4** Abundance of proteins in different *prt6* alleles.
**Fig. S5** Cruciferin peptides identified by TMT‐TAILS.
**Fig. S6** Activity‐based protein profiling probe specificity.
**Fig. S7** ERF‐dependence of protease activity.
**Fig. S8** ERF‐dependence of RD21 abundance.
**Table S1** Primers used in this study
**Methods S1** TMT labelling and enrichment of N‐termini by TAILS.
**Methods S2** Immunoblotting.

**Table S2** Identification of protein N‐termini with TMT‐TAILS

**Table S3** Quantification of peptides using TMT‐TAILS
