# Supplementary material for: N‐terminomics reveals control of Arabidopsis seed storage proteins and proteases by the Arg/N‐end rule pathway
Source: New Phytol. 2017 Nov 23;218(3):1106–26. doi: 10.1111/nph.14909 (PMC5947142; doi:10.1111/nph.14909)
Supplement: Supplementary file 1 — Fig. S1 Overview of the plant N‐end rule pathway. Fig. S2 The Arg/N‐end rule is active in etiolated seedlings. Fig. S3 Analysis of peptide quantification in Col‐0 and prt6. Fig. S4 Abundance of proteins in different prt6 alleles. Fig. S5 Cruciferin peptides identified by TMT‐TAILS. Fig. S6 Activity‐based protein profiling probe specificity. Fig. S7 ERF‐dependence of protease activity. Fig. S8 ERF‐dependence of RD21 abundance. Table S1 Primers used in this study Methods S1 TMT labelling and enrichment of N‐termini by TAILS. Methods S2 Immunoblotting. [file NPH-218-1106-s001.pdf]

*New Phytologist* **Supporting Information**

Article title: **N-terminomics reveals control of Arabidopsis seed storage proteins and proteases by the Arg/N-end rule pathway.**

Authors: Hongtao Zhang, Lucy Gannon, Kirsty L. Hassall, Michael J. Deery, Daniel J. Gibbs, Michael J. Holdsworth, Renier A.L. van der Hoorn, Kathryn S. Lilley, Frederica L. Theodoulou

Article acceptance date: 23 October 2017

The following Supporting Information is available for this article:

**Fig. S1** Overview of the plant N-end rule pathway

**Fig. S2** The Arg/N-end rule is active in etiolated seedlings

**Fig. S3** Analysis of peptide quantitation in Col-0 and *prt6*

**Fig. S4** Abundance of proteins in different *prt6* alleles

**Fig. S5** Cruciferin peptides identified by TMT™-TAILS

**Fig. S6** Activity-based protein profiling probe specificity

**Fig. S7** ERF-dependence of protease activity

**Fig. S8** ERF-dependence of RD21 abundance

**Table S1** Primers used in this study

**Table S2** Identification of protein N-termini with TMT™-TAILS

**Table S3** Quantification of peptides using TMT™-TAILS

**Methods S1** Tandem Mass Tag (TMT) labelling and enrichment of N-termini by TAILS

**Methods S2** Immunoblotting

**Figure S1. Overview of the plant N-end rule pathway**

Modified after Gibbs et al. (2014) and Zhang et al. (2015). The Arg/N-end rule pathway recognises non-acetylated, neo-N-termini generated by endopeptidase (EPase) cleavage of proteins to reveal primary destabilising residues (R,K,H,F,Y,W,L,I). Arabidopsis has at least three distinct Arg/N-end rule E3 ligases (N-recognins): PRT6 and PRT1 which recognise basic and aromatic N-termini, respectively and a genetically defined E3 ligase that recognises N-termini initiating with L and I for which the gene has not yet been identified (Garzón et al., 2007). The E3 ligases direct proteins to the ubiquitin proteasome (UPS) for degradation. Acidic N-terminal amino acids (D,E) are defined as secondary destabilising residues, since they can be post-translationally arginylated by the action of arginyl t-RNA transferase (ATE) enzymes. Nt amides (N,Q) are tertiary destabilising residues, and enter the Arg/N-end rule following enzymatic conversion to D and E, respectively by N-terminal amidases. Nt-cysteine, revealed either by methionine aminopeptidases (MetAPs) or EPases is also a tertiary destabilising residue. Nt-cysteine is subject to arginylation, following oxidation catalysed by plant cysteine oxidases (PCO) in the presence of NO. The proposed degradation of proteins initiating MΦ is based on data from yeast [Kim, H.K., Kim, R.R., Oh, J.H., Cho, H., Varshavsky, A. and Hwang, C.S. (2014) The N-terminal methionine of cellular proteins as a degradation signal. Cell. 156: 158-169] and the E3 ligase(s) responsible have not yet been identified. Targeting of proteins for degradation via the Pro/N-end rule, recently discovered in yeast [Chen, S.J., Wu, X., Wadas, B., Oh, J.H. and Varshavsky, A. (2017) An N-end rule pathway that recognizes proline and destroys gluconeogenic enzymes. Science. 355, eaal3655] has not been experimentally established in plants but is predicted to occur, based on the presence of E3 ligase subunits homologous to those of the GID complex. The Ac/N-end rule pathway recognises N-terminal acetylated residues. MetAPs cleave adjacent to small amino acid residues at position 2 (A,G,V,S,T,C,) to produce substrates for N-terminal acetyl transferases (NATs). Nt M can also be acetylated. Acetylated proteins are generally stable in the correctly folded and oligomeric state, but misfolding or complex misassembly may reveal Nt degrons which are recognised by Ac/N-recognins, leading to degradation by the ubiquitin proteasome system (UPS). Arabidopsis has homologues of the yeast Ac/N-recognins Doa10 and Not4.

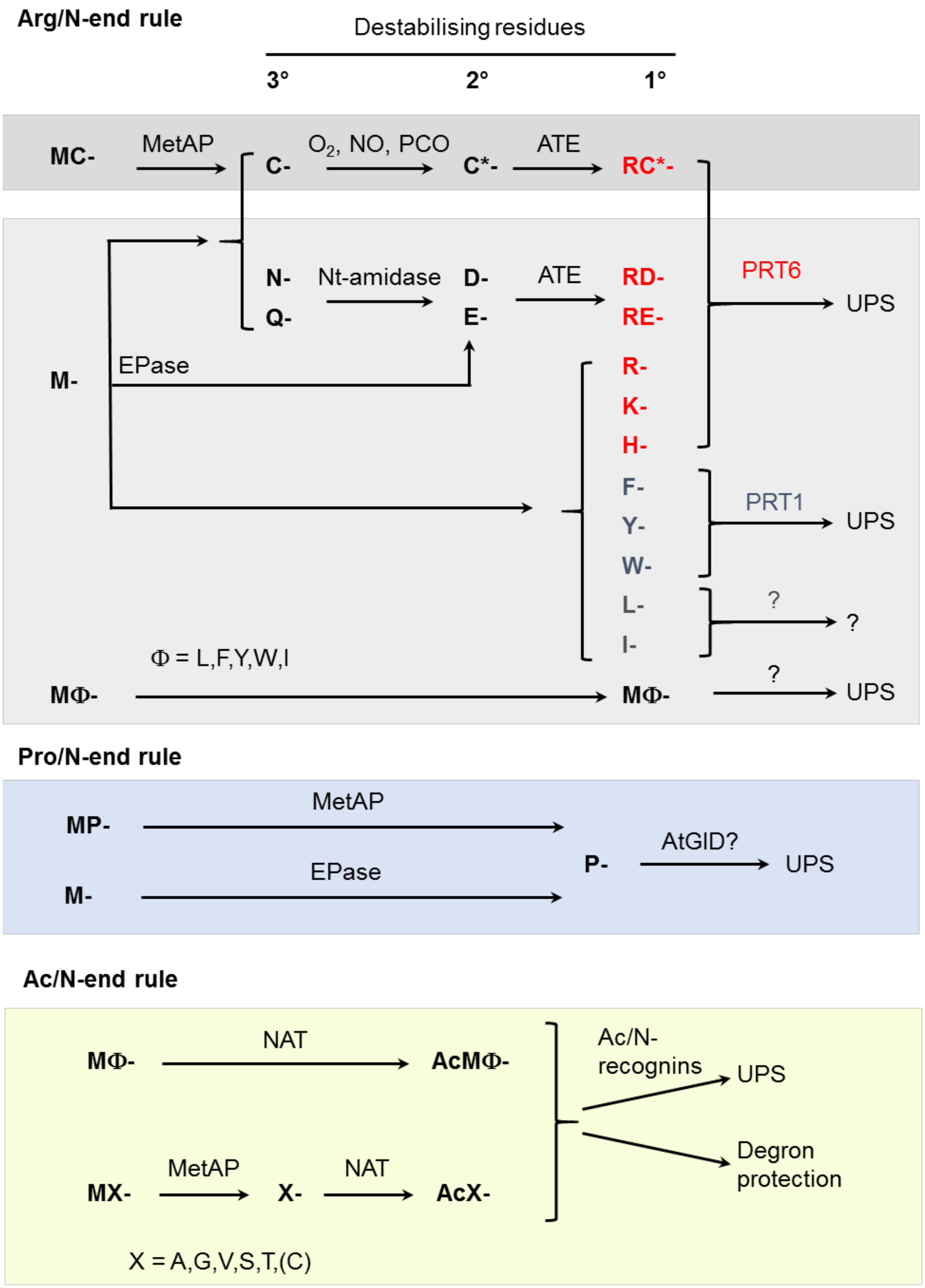

Arg/N-end rule
Destabilising residues
3°
2°
1°
MC-
MetAP
C-
O2, NO, PCO
C*-
ATE
RC*-
N-
Q-
Nt-amidase
D-
E-
ATE
RD-
RE-
PRT6
UPS
M-
EPase
R-
K-
H-
F-
Y-
W-
PRT1
UPS
L-
I-
?
?
Φ = L,F,Y,W,I
MΦ-
MΦ-
?
UPS
Pro/N-end rule
MP-
MetAP
M-
EPase
P-
AtGID?
UPS
Ac/N-end rule
MΦ-
NAT
AcMΦ-
MX-
MetAP
X-
NAT
AcX-
X = A,G,V,S,T,(C)
Ac/N-recognins
UPS
Degron protection

**Figure S2. The Arg/N-end rule is active in etiolated seedlings**

Expression of R-GUS protein stability reporter (Garzón et al., 2007) in 4 d old Col-0 and *prt6* etiolated seedlings. The stable control M-GUS is shown for comparison. The cartoon shows a schematic of the cleavable reporter. *In planta*, the fusion protein is cleaved by ubiquitin-specific proteases (indicated by arrow) to remove dihydrofolate reductase-ubiquitin (DHFR-Ub), generating a variant of GUS (light blue box) that is preceded by an unstructured region (light yellow box) with an Nt R residue and lysine residues (K) that can be ubiquitylated.

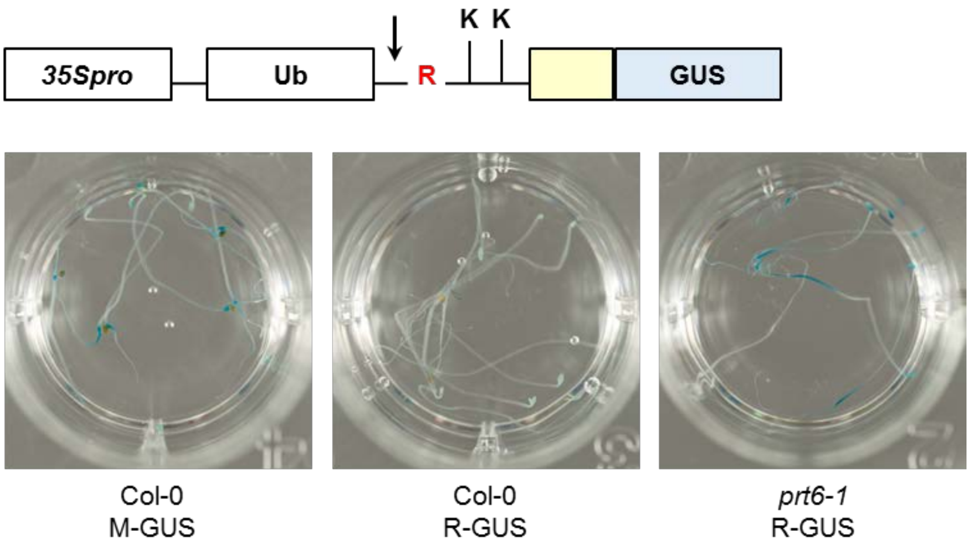

**Figure S3. Analysis of peptide quantitation in Col-0 and *prt6***

(a,b) Heat maps showing normalised peptide abundance for three biological replicates (1,2,3) digested with Trypsin and GluC, respectively. Each line represents an individual peptide; peptide abundance is indicated by the colour code to the right of the maps. (c,d) Volcano plots showing the relationship between statistical significance (p-value) on the y-axis and the biological significance (log2 fold change) on the x-axis. (e) Relative abundance of individual peptides in *prt6* relative to Col-0.

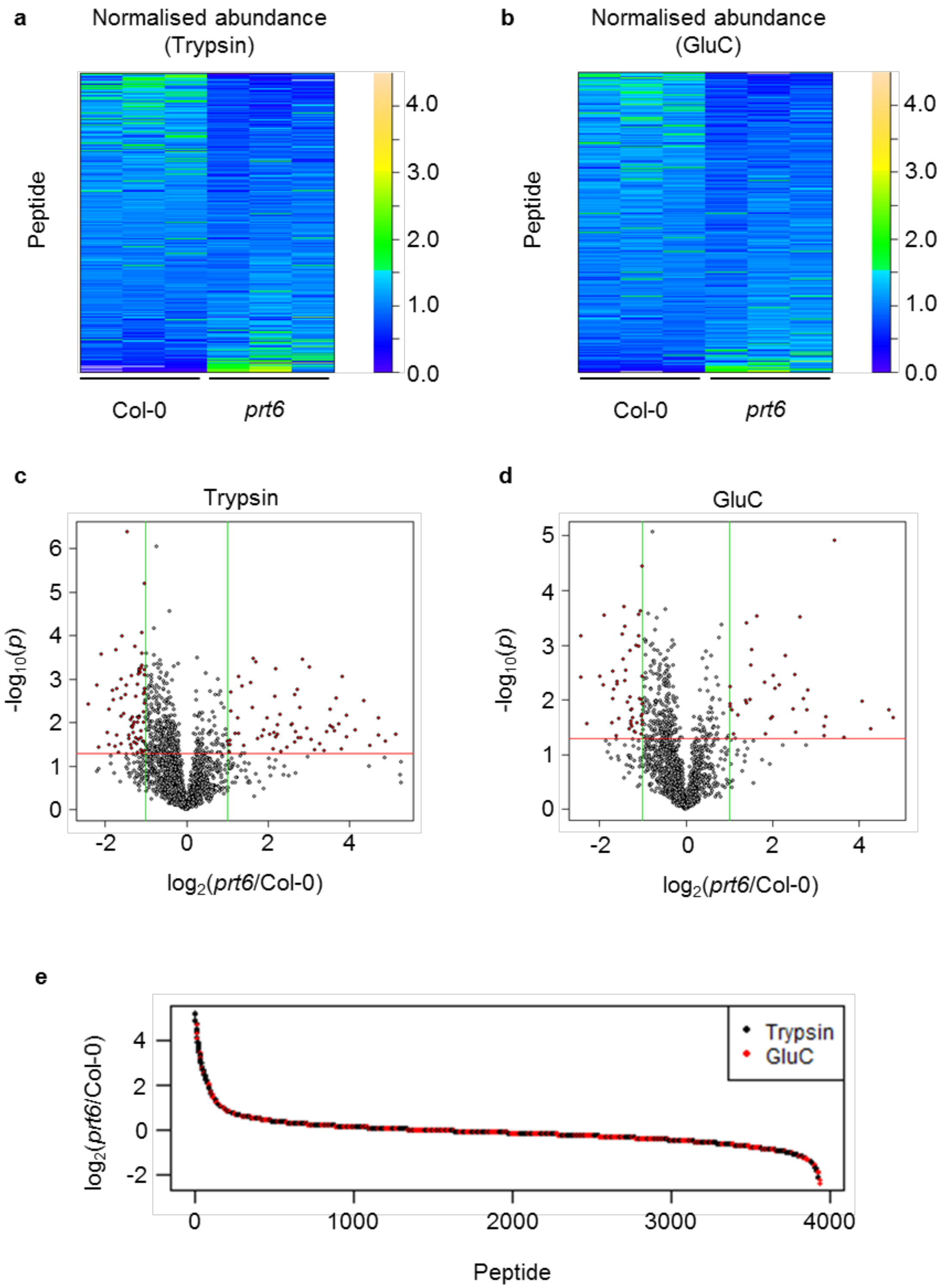

a
Normalised abundance
(Trypsin)
Peptide
4.0
3.0
2.0
1.0
0.0
Col-0
prt6
b
Normalised abundance
(GluC)
Peptide
4.0
3.0
2.0
1.0
0.0
Col-0
prt6
c
Trypsin
-log10(p)
log2(prt6/Col-0)
d
GluC
-log10(p)
log2(prt6/Col-0)
e
Trypsin
GluC
log2(prt6/Col-0)
Peptide

**Figure S4. Abundance of proteins in different *prt6* alleles**

A. Proteins were extracted from 4 d old etiolated seedlings and subjected to immunoblotting (25 μg/lane) with antisera towards pyruvate decarboxylase (PDC), alcohol dehydrogenase (ADH), cruciferin α subunit (α-Cru) and oleosin1 (Ole1). The left and right panels represent two independent experiments.

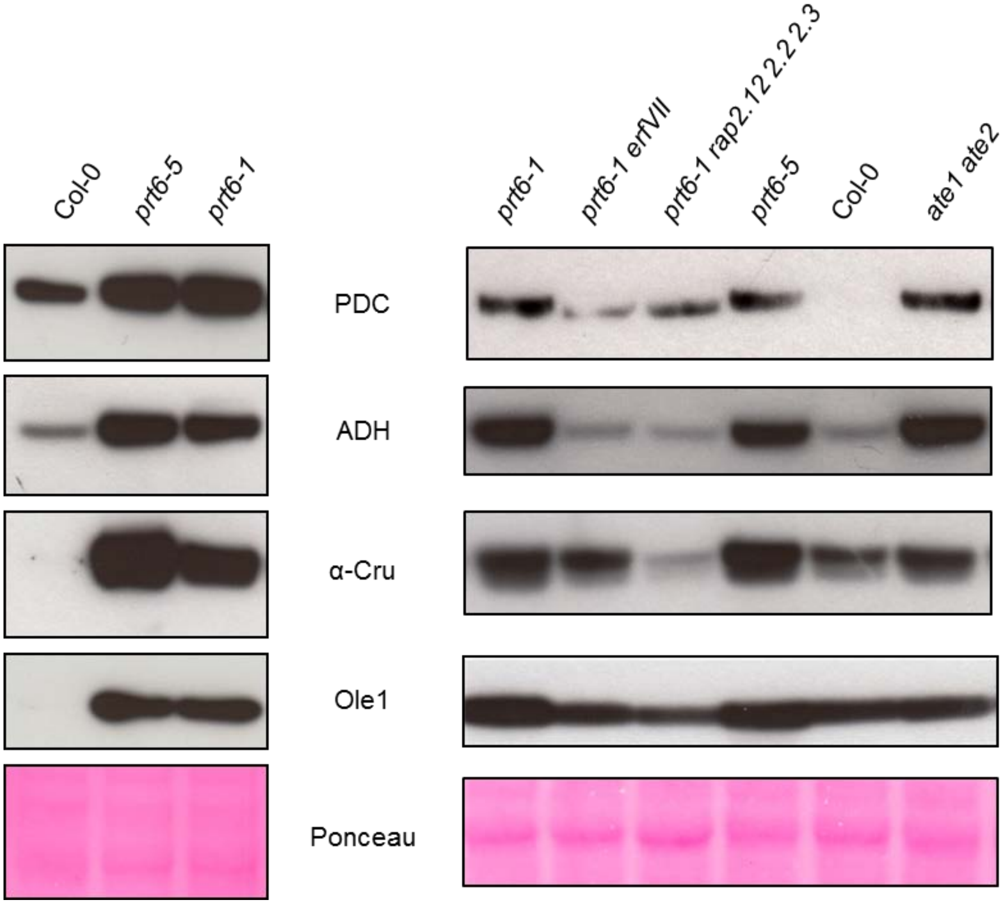

**Figure S5. Cruciferin peptides identified by TMT™-TAILS**

Graphs show the relative abundance of bona fide Nt peptides in *prt6*-5 (data are restricted to peptides increased >2-fold in *prt6* with $p<0.05$). Start positions are defined relative to the proproteins in the following gene models: At12S1/CRU3, At4g28520; At12S2, At1g03890; At12S3/CRU2, At1g03880; At12S4/CRU1, At5g44120. Peptides corresponding to N-termini of the α-and β-subunits (Higashi et al., 2006) are indicated in black and red, respectively. NB. some peptides were identified and quantified more than once. Quantification information for individual peptides is shown in Table S3.

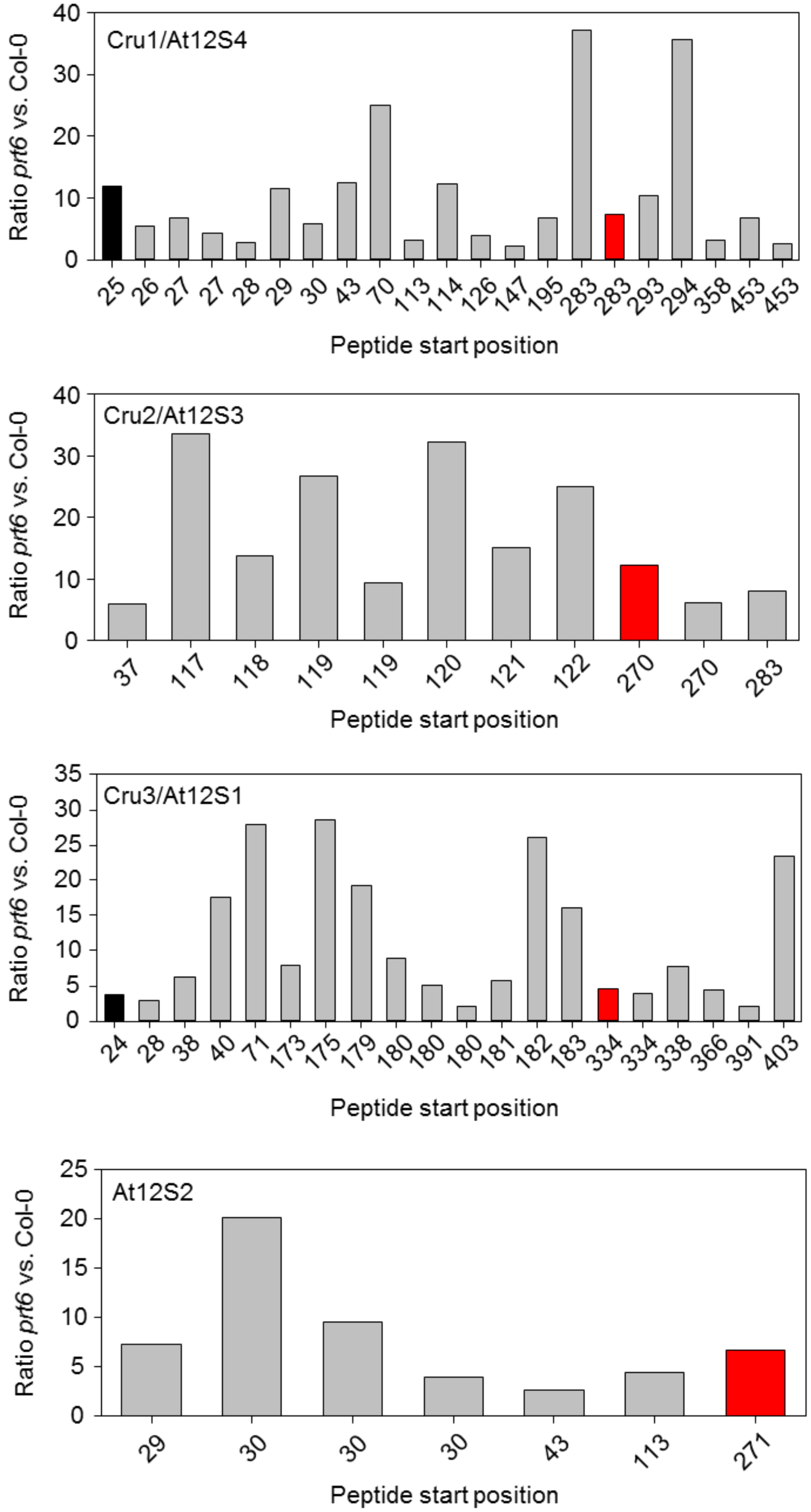
Cru1/At12S4
Ratio prt6 vs. Col-0
0
10
20
30
40
25 26 27 27 28 29 30 43 70 113 114 126 147 195 283 283 293 294 358 453 453
Peptide start position
Cru2/At12S3
Ratio prt6 vs. Col-0
0
10
20
30
40
37 117 118 119 119 120 121 122 270 270 283
Peptide start position
Cru3/At12S1
Ratio prt6 vs. Col-0
0
5
10
15
20
25
30
35
24 28 38 40 71 173 175 179 180 180 180 181 182 183 334 334 338 366 391 403
Peptide start position
At12S2
Ratio prt6 vs. Col-0
0
5
10
15
20
25
29 30 30 30 43 113 271
Peptide start position

**Figure S6. Activity-based protein profiling probe specificity**

(a) Protein extracts from 4 d old etiolated wild type and mutant seedlings were combined and incubated with probes MV201, FY01 and JOGDA1 in the presence and absence of the inhibitor, E64. Proteins were separated by SDS-PAGE and probes detected by fluorography. (b-d) Protein extracts from 4 d old etiolated Col-0, *prt6-5* and *ate1 ate2* seedlings were incubated with probes FY01 (b), MV201 (c) and JOGDA1 (d) and processed as in (a). Lanes showing mixed sample extract incubated in the absence of probe, or with probe plus inhibitor are included as negative controls. Arrowheads indicate specific binding. The positions of $M_r$ markers (kDa) are indicated at the left-hand side of each panel.

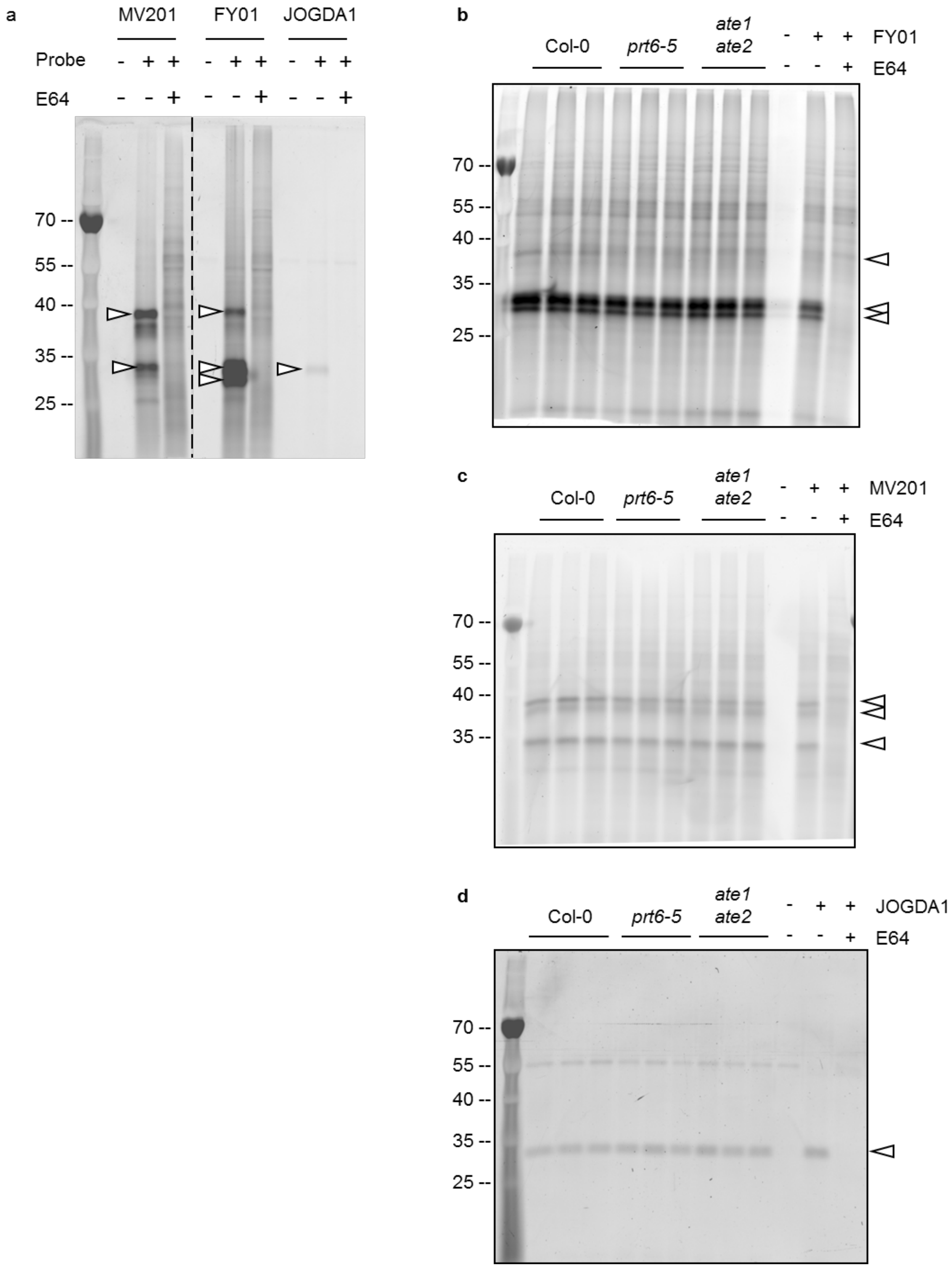
a
MV201
FY01
JOGDA1
Probe
E64
70
55
40
35
25
b
Col-0
prt6-5
ate1
ate2
FY01
E64
70
55
40
35
25
c
Col-0
prt6-5
ate1
ate2
MV201
E64
70
55
40
35
d
Col-0
prt6-5
ate1
ate2
JOGDA1
E64
70
55
40
35
25

**Figure S7. ERF-dependence of protease activity**

Gel images used for quantification in Fig. 7. Protein extracts from 4 d old etiolated N-end rule and *erfVII* combination mutant seedlings were incubated with probes (a) FY01 RD21; aleurin-like proteases; ALPs), (b) MV201 (RD21) and (c) JOGDA1 (Cathepsin B; AtCathB). Proteins were separated by SDS-PAGE and probes detected by fluorography. Three biological replicates were used for each genotype. Arrowheads indicate specific binding. The positions of $M_r$ markers (kDa) are indicated at the left-hand side of each panel. Panel (d) shows quantification for MV201 RD21 signal in (b). Values are means $\pm$ SE (n=3); ** = $p<0.01$; *** = $p<0.001$.

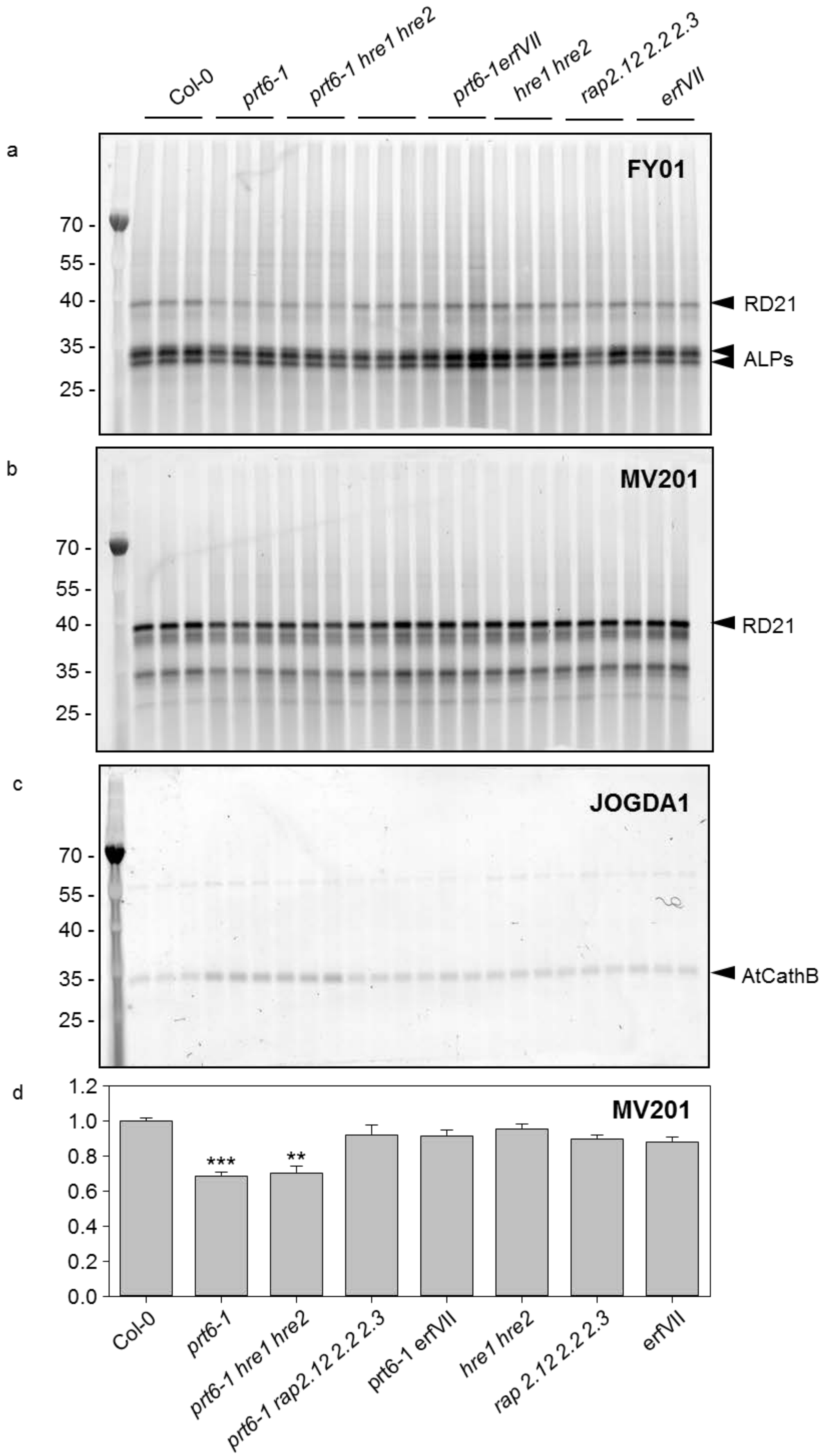

Col-0
prt6-1
prt6-1 hre1 hre2
prt6-1erfVII
hre1 hre2
rap2.12 2.2 2.3
erfVII
a
FY01
70 -
55 -
40 -
35 -
25 -
RD21
ALPs
b
MV201
70 -
55 -
40 -
35 -
25 -
RD21
c
JOGDA1
70 -
55 -
40 -
35 -
25 -
AtCathB
d
MV201
1.2
1.0
0.8
0.6
0.4
0.2
0.0
***
**
Col-0
prt6-1
prt6-1 hre1 hre2
prt6-1 rap2.12 2.2 2.3
prt6-1 erfVII
hre1 hre2
rap 2.12 2.2 2.3
erfVII

**Fig. S8 ERF-dependence of RD21 abundance**

Immunoblots probed with antisera raised to RD21A. Protein extracts from equal numbers of 3 d or 4 d old etiolated seedlings were loaded in each lane.

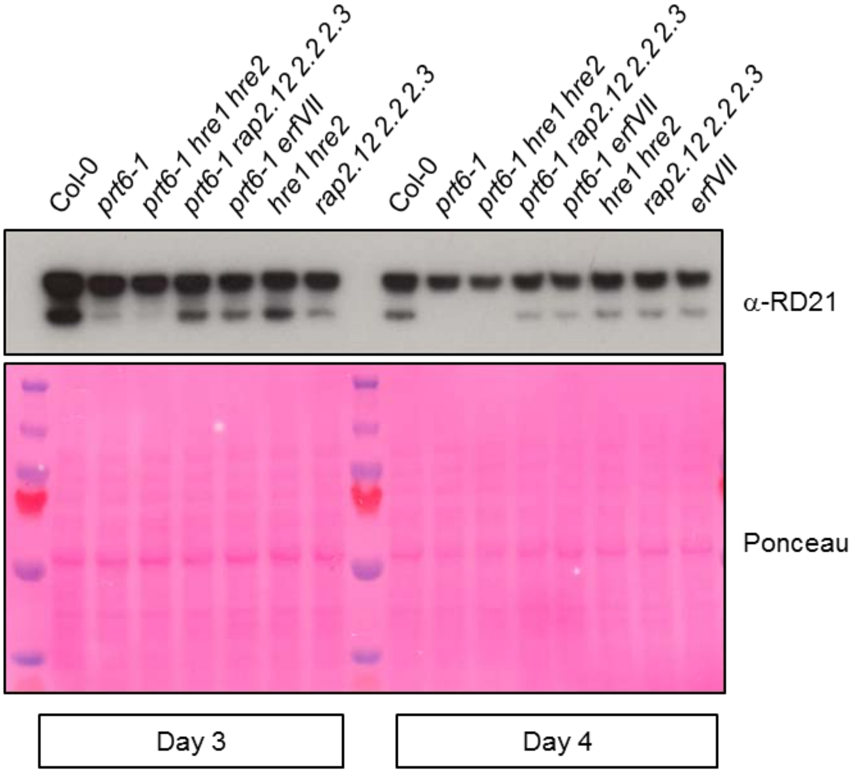


**Table S1** Primers used in this study

| Primer | Sequence 5'-3' |
|---|---|
| ACT2F | ccgctctttctttccaagc |
| ACT2R | ccggtaccattgtcacacac |
| TUB4F | gctgagaacagcgattgtctt |
| TUB4R | agttcccattccagatccagt |
| PYL2F | aggtttacacggtggttcttg |
| PYL2R | gacgacagtgtccacaaacatt |
| CTB-1F | ttggctggggaacttctg |
| CTB-1R | tcctgatcttgaagtaaccatcat |
| CTB-2F | cttagatcagggtcactgtggtt |
| CTB-2R | gatcattgacagataaagaaacattca |
| CTB-3F | gaagattattggttgatggcaaa |
| CTB-3R | tcctctccttatcatgaaataacca |

| | |
|---|---|
| CYSB_139_F | ttctcgaggcaggacagaag |
| CYSB_139_R | ttcaaccacggcttgacc |
| RD21AF | tgcgttccagctctatgattc |
| RD21AR | cccatgagtttctcacaatcc |
| SLP2F | gttataaggacggcgacgaa |
| SLP2R | ctctatcctcgcccgataaa |
| RD19F | ttactcccgtcaaaaatcagg |
| RD19R | caccttccaaagctccagtg |
| CP1_94F | gaggttccagagacggttga |
| CP1_94R | aaaacgcccaacaacttcc |

**Table S2** Identification of protein N-termini with TMT™-TAILS (separate Excel file)

**Table S3** Quantification of peptides using TMT™-TAILS (separate Excel file)

**Methods S1 Tandem Mass Tag (TMT) labelling and enrichment of N-termini by TAILS**

Key steps in the TAILS procedure are: (1) chemical labelling of protein N-termini (and lysine ε-amino groups) using different isotopic versions of amino-reactive TMT reagents for control and test samples, (2) digestion of the labelled protein samples with two orthogonal proteases to generate internal peptides with free amino groups. Here, trypsin, a well documented endopeptidase was used in the knowledge that any cleavage of N-terminal arginines is likely to be insignificant. GluC was selected as having a distinct cleavage site (3) enrichment of original N-terminal peptides by negative selection using an amine-reactive matrix to remove internal peptides, (4) separation using high pH reverse phase fractionation and (5) identification of peptides by tandem MS and relative quantification of peptides in test and control samples using isotopic ratios (Kleifeld et al., 2010, 2011; Zhang et al., 2015). Arabidopsis proteins were extracted in 6 M Guanidine hydrochloride GuHCl), 100 mM HEPES (pH 7.5), containing Complete Mini protease inhibitor Cocktail, PhosSTOP Phosphatase Inhibitor Cocktail (Roche) and 100 µmol/l MG-132 (Sigma). Protein aliquots (200 µg) were methanol/chloroform precipitated, re-dissolved in 30 µl 6M GuHCl and adjusted to 100 µl by adding 50 µl filtered water and 20 µl 1M HEPES, pH 8.0. Reduction was done by adding 5µl 0.5M TCEP (Tris[2-carboxyethyl] phosphine) in 200 mM

TEAB (triethylammonium bicarbonate) pH 8.0 and incubated for 60 min at 55°C. Samples were alkylated by adding 7.5 µl of 375 mM iodoacetamide in 100 mM TEAB and incubating at 25°C in the dark. Proteins were individually labelled by whole protein isobaric labelling using TMT™6-plex reagents (Thermo Scientific, Waltham, MA), with multiple labels as biological replicates (Col-0 labelled with $TMT^6$-127, -129, 131; *prt6-5* with $TMT^6$-126, -128, -130). Equal amounts of proteins were mixed and methanol/chloroform precipitated, re-dissolved in 250 µl 50 mM HEPES Ph 7.5. After Trypsin (1:50) or GluC (1:50) digestion, aliquots of protein were subjected to LC-MS/MS ("preTAILS analysis"; Lange et al., 2014). Aliquots of 500 µg TMT-labelled and N-terminally blocked peptides were negatively enriched by selective removal of the internal peptides, via 2 mg aldehyde-functionalized dendritic polymer (http://flintbox.com/public/project/1948/). Enriched N-termini were desalted using Sep-Pack light C18 (Waters). Peptides corresponding to one third of each sample were separated by high pH reverse-phase chromatography using a Waters reverse-phase nano column as described in Zhang et al. (2015). 40 fractions were collected and selected fractions were combined to ensure a good range of hydrophobicities for each LC-MS/MS run. 20 and 17 combined fractions were injected for Trypsin and GluC, respectively. One third of each sample was also used for a single 3 h run without high pH fractionation.

All LC-MS/MS experiments were performed using a Dionex Ultimate 3000 RSLC nanoUPLC (Thermo Fisher Scientific Inc, Waltham, MA, USA) system and a QExactive Orbitrap mass spectrometer (Thermo Fisher Scientific Inc, Waltham, MA, USA). Separation of peptides was performed by reverse-phase chromatography at a flow rate of 300 nl/min and a Thermo Scientific reverse-phase nano Easy-spray column (Thermo Scientific PepMap C18, 2 µm particle size, 100A pore size, 75 µm i.d. x 50cm length). Peptides were loaded onto a pre-column (Thermo Scientific PepMap 100 C18, 5 µm particle size, 100 A pore size, 300 µm i.d. x 5 mm length) from the Ultimate 3000 autosampler with 0.1 % formic acid for 3 minutes at a flow rate of 10 µl/min. After this period, the column valve was switched to allow elution of peptides from the pre-column onto the analytical column. Solvent A was water + 0.1 % formic acid and solvent B was 80 % acetonitrile, 20 % water + 0.1 % formic acid. The linear gradient employed was 4-140 B in 100

minutes (the total run time including column washing and re-equilibration was 120 minutes).

The LC eluent was sprayed into the mass spectrometer by means of an Easy-spray source (Thermo Fisher Scientific Inc.). All *m/z* values of eluting ions were measured in an Orbitrap mass analyzer, set at a resolution of 70000. Data-dependent scans (Top 20) were employed to automatically isolate and generate fragment ions by higher energy collisional dissociation (HCD) in the quadrupole mass analyser and HCD cell respectively. Measurement of the resulting fragment ions was performed in the Orbitrap analyser, set at a resolution of 17500. Peptide ions with charge states of between 2+ and 5+ were selected for fragmentation.

**Methods S2 Immunoblotting**

Proteins were quantified using Bradford's reagent and separated in pre-cast 4-12% Bis-Tris gels (TermoFisher), using 1 X SDS MES buffer. Proteins were stained with Coomassie Brilliant Blue or transferred to PVDF using iblot dry blotting system (ThermoFisher). Protein transfer was confirmed by Ponceau S staining. Blots were blocked with 5 % milk in Tris buffered saline with 0.05 % tween 20 (TBST) for least 1 h, followed by a brief rinse in TBST and incubated with primary antibodies overnight at 4 °C. The primary antibodies are used at the following dilutions: *Brassica napus* Cruciferin (Wan et al., 2007), 1:10,000 to 20,000; OLE1 (anti-rS3; D'Andrea et al. 2007), 1:5,000 to 10,000; Anti N-terminal AtCathB3 (a kind gift of Dr Patrick Gallois, University of Manchester), 1:1000; Arabidopsis RD21 (residues 137 – 150; LPESIDWRKKGAVAC; Kaschani et al. 2009, kind gift of Prof Carol Mackintosh, Dundee), 1:1000; PDC and ADH (Agrisera, Vännäs, Sweden) 1:10,000 and 1:3,000, in blocking buffer respectively. Following washing three times with TBST for 10 min, blots were incubated with secondary antibody (anti rabbit horseradish peroxidase conjugate A0545, Sigma), diluted 1: 25,000 in blocking buffer or for RD21 with rabbit anti-Sheep IgG Antibody, HRP conjugate 1: 5,000 (12-342, Sigma). Blots were then washed and developed with ECL reagent SuperSignal™ West Pico Chemiluminescent Substrate (AtCathB3 and RD21) or Femto Maximum Sensitivity Substrate (ADH, PDC, α-Cruciferin and Ole1) (ThermoFisher).

**Supporting references**


**Chen SJ, Wu X, Wadas B, Oh JH, Varshavsky A.** (2017) An N-end rule pathway that recognizes proline and destroys gluconeogenic enzymes. Science. **355**: eaal3655.

**D'Andréa S, Jolivet P, Boulard C, Larré C, Froissard M, Chardot T.** (2007) Selective one-step extraction of *Arabidopsis thaliana* seed oleosins using organic solvents. *J Agric Food Chem.* **55**: 10008-10015.

**Garzón M, Eifler K, Faust A, Scheel H, Hofmann K, Koncz C, Yephremov A, Bachmair A.** (2007) *PRT6*/At5g02310 encodes an Arabidopsis ubiquitin ligase of the N-end rule pathway with arginine specificity and is not the *CER3* locus. *FEBS Lett*. **581**: 3189-3196.

**Gibbs DJ, Bacardit J, Bachmair A, Holdsworth MJ** (2014) The eukaryotic N-end rule pathway: conserved mechanisms and diverse functions. *Trends Cell Biol*. **24**: 603-611.

**Higashi Y, Hirai MY, Fujiwara T, Naito S, Noji M, Saito S.** (2006) Proteomic and transcriptomic analysis of Arabidopsis seeds: molecular evidence for successive processing of seed proteins and its implication in the stress response to sulphur nutrition. *Plant J.* **48**: 557-571.

**Kaschani F, Verhelst SH, van Swieten PF, Verdoes M, Wong CS, Wang Z, Kaiser M, Overkleeft HS, Bogyo M, van der Hoorn RA.** (2009) Minitags for small molecules: detecting targets of reactive small molecules in living plant tissues using 'click chemistry'. *Plant J*. **57**: 373-85.

**Kim HK, Kim RR, Oh JH, Cho H, Varshavsky A, Hwang CS.** (2014) The N-terminal methionine of cellular proteins as a degradation signal. Cell **156**: 158-169.

**Kleifeld O, Doucet A, auf dem Keller U, Prudova A, Schilling O, Kainthan RK, Starr AE, Foster LJ, Kizhakkedathu JN, Overall CM.** (2010) Isotopic labeling of terminal amines in complex samples identifies protein N-termini and protease cleavage products. *Nat Biotechnol.* **28**: 281-288.

**Kleifeld O, Doucet A, Prudova A, auf dem Keller U, Gioia M et al.** (2011) Identifying and quantifying proteolytic events and the natural N terminome by terminal amine isotopic labeling of substrates. *Nat Protoc*. **6**: 1578-1611.

**Wan L, Ross AR, Yang J, Hegedus DD, Kermode AR.** (2007) Phosphorylation of the 12 S globulin cruciferin in wild-type and *abi1-1* mutant *Arabidopsis thaliana* (thale cress) seeds. *Biochem J.* **404:** 247-256.

**Zhang H, Deery MJ, Gannon L, Powers SJ, Lilley KS, Theodoulou FL.** (2015) Quantitative proteomics analysis of the Arg/N-end rule pathway of targeted degradation in Arabidopsis roots. *Proteomics* **15**: 2447-2457.
